# The R451 site is critical for PTPN18 to exert tumor suppressive effects in breast cancer through the negative regulatory interacting protein fibrillarin

**DOI:** 10.1038/s41419-025-08395-1

**Published:** 2026-01-20

**Authors:** Na Zhang, Tao Wang, Bin Bai, Xiaonan Zhang, Wenying Xu, Weilu Chen, Yang Yu, Bing Wang

**Affiliations:** 1 Institute of Biochemistry and Molecular Biology, College of Life and Health Sciences, Northeastern University, #195 Chuangxin Road, Hunnan Xinqu, Shenyang, Liaoning 110169 China; 2 Key Laboratory of Bioresource Research and Development of Liaoning Province, College of Life and Health Sciences, Northeastern University, Shenyang, #195 Chuangxin Road, Hunnan Xinqu, Shenyang, Liaoning 110169 China; 3 Research Laboratory Center, Guizhou Provincial People’s Hospital, Nanming, Guiyang, 550002 Guizhou P.R. China; 4 Department of Dermatology, The First Hospital of China Medical University, Shenyang, Liaoning 110001 China; 5 Department of Pathophysiology, Bengbu Medical University, Longzihu, Bengbu, 233030 Anhui P.R. China

**Keywords:** Breast cancer, Phosphorylation, Post-translational modifications, Cell growth

## Abstract

PTPN18 is a member of the PEST (proline-glutamic acid-serine-threonine rich sequence) protein tyrosine phosphatase subfamily that has been intensively studied in immune cells. Here, we identified a novel PTPN18-interacting protein, fibrillarin (FBL), through mass spectrometry analysis and clarified the binding sites and interaction motifs via peptide mapping. The R451 site of PTPN18 and the V187 site of FBL dominate the interaction between PTPN18 and FBL. Further studies suggest that PTPN18, but not PTPN18 R451A, can dephosphorylate the Y313 site of FBL and can reduce the protein expression level of FBL by promoting its ubiquitin proteasome degradation. In addition, PTPN18 can affect its downstream functions, including the MAPK signaling pathway and methylation of rRNA 2′-O and histone H2AQ104 sites, as well as RNA synthesis through negative regulation of FBL, whereas PTPN18 R451A cannot. As a result, the interaction between PTPN18 and FBL affects the proliferation and apoptosis of breast cancer cells, thus inhibiting tumor growth. This study reveals a novel mechanism through which PTPN18 inhibits breast cancer progression and further refines the PTPN18 protein interaction network, which is important for understanding its role in cell signaling, revealing disease mechanisms, discovering new drug targets, and developing new treatments.

## Introduction

Breast cancer is the leading cause of cancer death among women in 112 countries worldwide [1], and elucidating the molecular mechanism of breast cancer development is the core prerequisite for conquering this disease. The protein tyrosine phosphatase nonreceptor type 18 (PTPN18) is an important cellular regulator that can regulate a variety of life activities of cells through interactions with different proteins [2–7]. Analysis of data from the Breast Cancer Integrative Platform (BCIP) database revealed that high PTPN18 expression was beneficial for the overall survival of patients with breast cancer [8]. Its N-terminal catalytic domain and C-terminal PEST domain regulate the phosphorylation level and protein level of HER2, respectively, and have important effects on the occurrence, development and prognosis of breast cancer [9–11]. Therefore, studying the molecular mechanism and function of PTPN18 can aid in the development of new breast cancer treatment strategies.

Human rRNA 2’-O-methyltransferase fibrillarin (FBL; NCBI Gene ID: 2091) is an abundant, phylogenetically conserved nucleolar protein [12–14] that plays a fundamental role in all major posttranscriptional activities of ribosome biogenesis, such as pre-rRNA processing, rRNA modification, and ribosome assembly [14–16]. FBL has methylation activity, which is responsible for the 2′-O-methylation (2′-O-Me) of ribosomal RNAs (rRNAs) and the methylation of histone H2A at glutamine 104 [17, 18]. FBL overexpression contributes to tumorigenesis and is associated with poor survival in patients with breast cancer [19].

Our previous studies revealed that PTPN18 can synergize with CSK to inhibit Src kinase-triggered signaling events and can inhibit MVP and importin β2-mediated breast cancer metastasis [2, 10]. To further explore the function of PTPN18 and elucidate its mechanism of action in breast cancer, this study first discovered and confirmed that PTPN18 can bind to a new substrate protein, FBL, by mass spectrometry and coimmunoprecipitation (Co-IP) and revealed the amino acid sites that dominate its interaction and the specific sites at which PTPN18 regulates the tyrosine phosphorylation of FBL. PTPN18 binds to FBL through the R451 site and regulates its phosphorylation and ubiquitination, which in turn affects the MAPK signaling pathway, methylation of rRNA 2′-O and histone H2AQ104 sites, and RNA synthesis, ultimately exerting antitumor effects in breast cancer. In this study, we identified and investigated a novel FBL-dependent critical mechanistic pathway for the antitumor activity of PTPN18. Dissection of the PTPN18 protein interaction network is essential for revealing cellular mechanisms and biological functions, contributing to the discovery of new drug targets and the development of new therapeutic approaches and opening new avenues for exploring the mechanisms of disease development and progression.

## Results

### PTPN18 interacts exogenously and endogenously with FBL

**Fig. 1:**
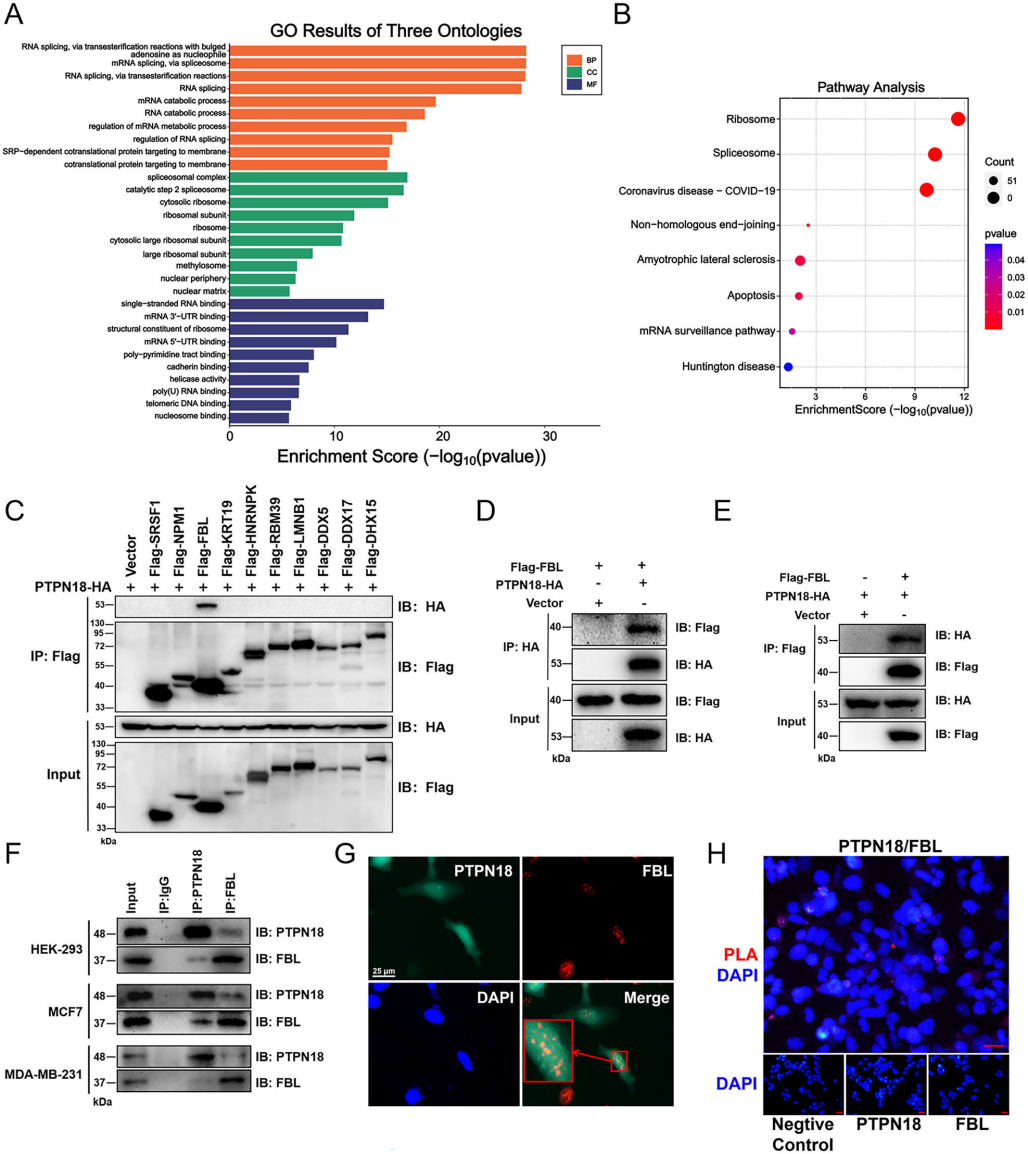
PTPN18 interacts exogenously and endogenously with FBL. **A** GO analysis was performed on proteins that may interact with PTPN18, as determined by IP‒MS. **B** KEGG enrichment analysis was performed on the identified proteins. **C** FBL, a protein capable of interacting with PTPN18, was selected for use in the Co-IP experiments in HEK293 cells. **D**, **E** Positive and reverse Co-IP was performed in MCF7 cells to verify the interaction of PTPN18 with FBL. **F** The physical interaction between endogenous PTPN18 and FBL was validated using IP in HEK293, MCF7 and MDA-MB-231 cells. **G** Representative immunofluorescence images showing the colocalization (orange) of PTPN18 (green) and FBL (red) in HEK293 cells. **H** A proximity ligation assay (PLA) was used to analyze the endogenous interaction of PTPN18 with FBL in MCF7 cells. The PLA signal is shown in red, and the nucleus is shown in blue. Negative controls were performed without the primary antibody; PTPN18 controls and FBL controls were performed using a single primary antibody. Fluorescence images were acquired using a 40X objective. Scale bar, 25 μm. All the experimental data were verified in at least three independent experiments (*n* ≥ 3).

Statistical analysis of the BCIP database revealed that high PTPN18 expression was beneficial for the overall survival of patients with breast cancer (Fig. S1A-D); therefore, determining its antitumor mechanism is necessary [8]. To achieve this goal, we first identified proteins that potentially interacted with PTPN18 in HEK293 cells by coimmunoprecipitation (Co-IP) and mass spectrometry (MS). GO (Fig. 1A) and KEGG (Fig. 1B) enrichment analyses were performed to compare the differentially expressed proteins between the experimental group and the control group, and the results revealed that PTPN18 was closely associated with RNA processing modification and ribosomes. Co-IP experiments were performed after coexpression in HEK293 cells to screen and validate the ability of the protein FBL to interact with PTPN18 (Fig. 1C). Because the identification of interacting proteins by mass spectrometry is affected by protein location, antibody interference, interaction strength, and protein stability, among other variables, the potential interactions of other failed validation candidate proteins can be explored in subsequent studies. In this paper, we preferred FBL as the research object. FBL has a stronger interaction with PTPN18 and a more consistent function according to the enrichment analysis results. As shown in Fig. 1D and E, both ectopically expressed exogenous proteins could interact in MCF cells regardless of whether HA or Flag was immunoprecipitated. Next, we further validated the interaction of endogenous PTPN18 and FBL proteins in the HEK293 and breast cancer cell lines MCF7 and MDA-MB-231, respectively (Fig. 1F). In addition, immunofluorescence colocalization and proximity ligation assays (PLAs) revealed the spatial association and physical contact of PTPN18 with FBL, respectively (Fig. 1G, H). The above experimental results revealed a stable interaction between PTPN18 and FBL.

### R451 of PTPN18 and V187 of FBL dominate the interaction between PTPN18 and FBL

**Fig. 2:**
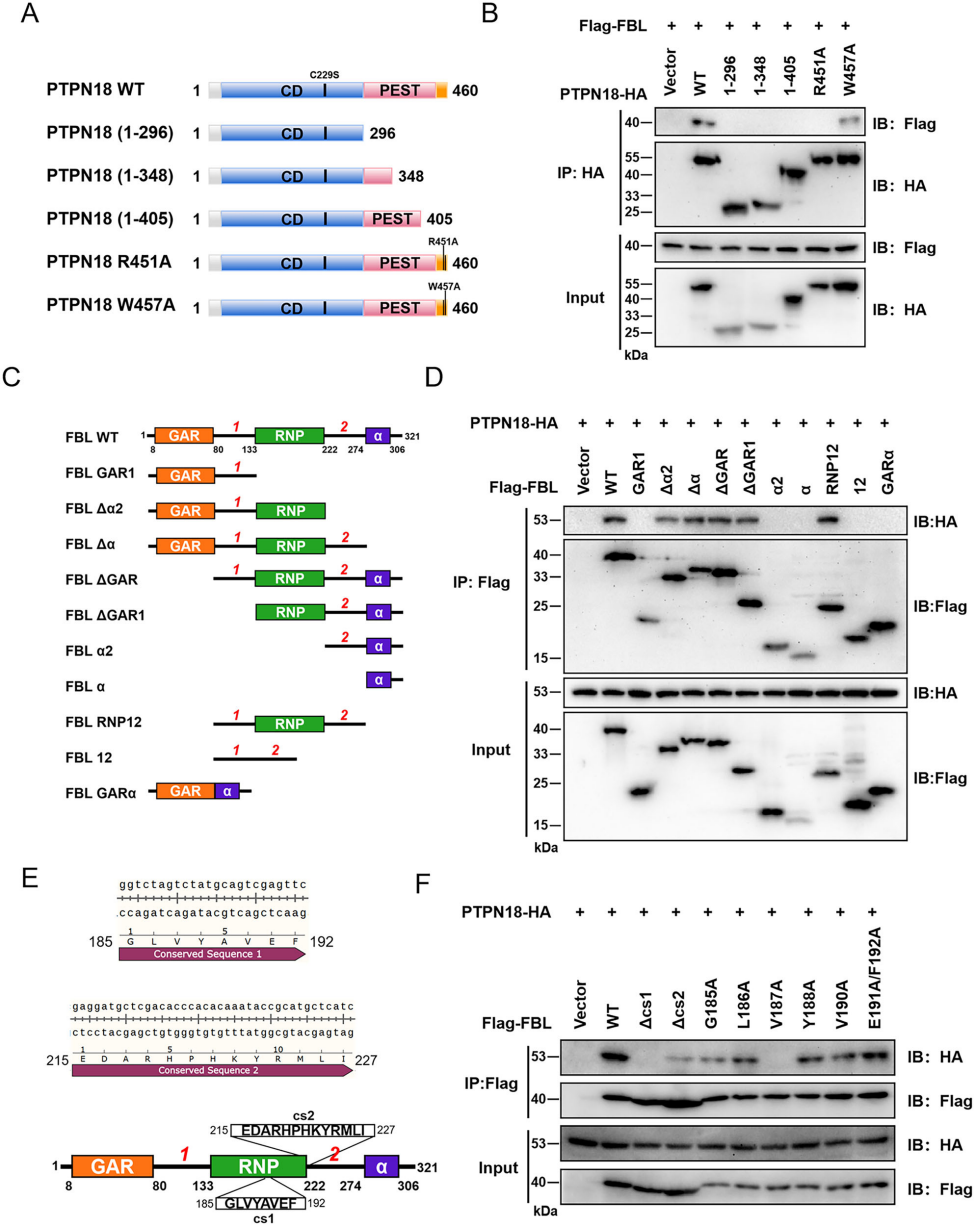
R451 of PTPN18 and V187 of FBL dominate the interaction between the two. **A** Schematic representation of different PTPN18 truncation fragments and point mutations. **B** Binding of different PTPN18 mutants to FBL detected by Co-IP in HEK293 cells. **C** Schematic representation of different FBL truncations on the basis of their functional domains. **D** Co-IP was used to detect the binding of different FBL mutants to PTPN18 in HEK293 cells. **E** Schematic representation of two conserved sequences of RNP domains of FBL. **F** Sites of the master control interacting with PTPN18 were searched for by FBL deletion mutation and point mutation in HEK293 cells. All the experimental data were verified in at least three independent experiments (*n* ≥ 3).

To identify the site at which PTPN18 binds to FBL, we cloned fragments of different domains of the PTPN18 gene (Fig. 2A). Co-IP experiments revealed that only a fragment containing 55 amino acids (406− 460) at the C-terminus of PTPN18 was able to bind to FBL. It was previously reported that both R444 and W450 (murine) mediate PTPN18 binding to PSTPIP2 [5]. Thus, we generated separate point mutations at the R451 and W457 (human) sites of PTPN18, and the Co-IP results revealed that R451 rather than W457 dominated the interaction between PTPN18 and FBL (Fig. 2B). We subsequently cloned truncated recombinant plasmids containing different FBL domains (Fig. 2C). The Co-IP results revealed that FBL truncations lacking the RNP domain were unable to maintain binding to PTPN18 (Fig. 2D). The RNP domain is composed mainly of one RNA-binding sequence [20] and two conserved sequences (Fig. 2E), and we speculated that these two conserved sequences were likely to mediate the interaction of FBL with PTPN18. We therefore performed deletion mutations in these two conserved sequences, and the results revealed that the presence or absence of conserved sequence 1 determined whether FBL could interact with PTPN18 (Fig. 2F). To further identify the amino acid sites on FBL that dominated the interaction with PTPN18, we performed alanine scanning mutagenesis of the conserved sequence 1. The results revealed that the interaction between FBL and PTPN18 disappeared when V187 was mutated to A (Fig. 2F). In summary, the experimental results revealed that R451 of PTPN18 and V187 of FBL dominate the interaction between the two.

### R451 is required for PTPN18 to regulate the phosphorylation of FBL via Y313

**Fig. 3:**
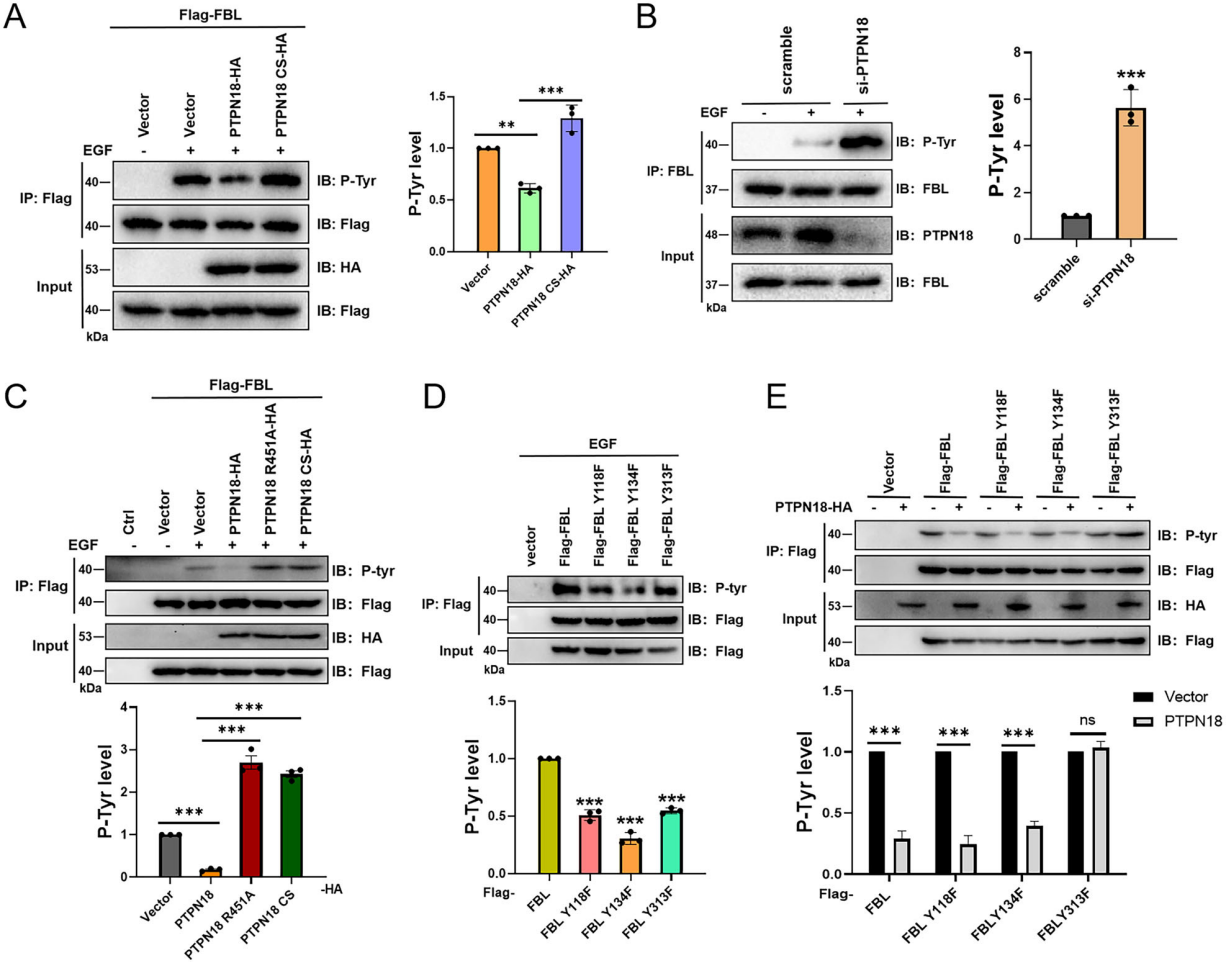
R451 is essential for PTPN18 to regulate the phosphorylation of FBL through Y313. **A** Co-IP was used to examine the effects of PTPN18 overexpression and PTPN18 CS on FBL tyrosine phosphorylation levels in MCF7 cells. Right: FBL tyrosine phosphorylation level statistics bar graph. **B** Knockdown of PTPN18 in MCF7 cells significantly increased tyrosine phosphorylation levels in FBL. Right: Statistical plots of phosphorylation levels. **C** Effect of interaction versus noninteraction (PTPN18 R451A) on FBL phosphorylation levels. Below is a statistical plot of P-tyr levels. **D** All three tyrosine residues of FBL could be phosphorylated in MCF7 cells. P-tyr statistics are shown below. **E** Phosphorylation at FBL Y313 is regulated by PTPN18. Below are the statistical figures. Representative results of three independent experiments are shown (*n* = 3). **P < 0.01; ***P < 0.001; ns, not significant.

The phosphorylation of tyrosine residues in proteins is closely associated with the etiology of many human diseases, such as cancer [21–23]. PTPN18 is involved as a protein tyrosine phosphatase that regulates a variety of life activities in cells; thus, we next investigated whether PTPN18 was able to regulate tyrosine phosphorylation levels in FBL. The optimal conditions for EGF stimulation of FBL tyrosine phosphorylation in MCF7 cells were 100 ng/ml for 30 min (Fig. S2A, B). PTPN18 overexpression reduced tyrosine phosphorylation levels in FBL, whereas PTPN18 C229S enzyme-inactivating mutations did not (Fig. 3A). Furthermore, knockdown of PTPN18 resulted in increased phosphorylation of FBL (Fig. 3B). Following the introduction of the PTPN18 R451A mutant, it was found that the dephosphorylation of FBL by PTPN18 required a combination of both (Fig. 3C). According to the DTU Health Tech-NetPhos-3.1 search, three tyrosine sites, Y118, Y134 and Y313, above the threshold might be dephosphorylated by PTPN18 in FBL (Fig. S2C). When either of these three sites was mutated, their phosphorylation levels were reduced to varying degrees compared with those without mutations, indicating that they could all be phosphorylated in MCF7 cells (Fig. 3D). However, only the phosphorylation level of FBL Y313F was essentially unaffected by PTPN18 overexpression, indicating that PTPN18 could regulate the Y313 phosphorylation site of FBL (Fig. 3E). Taken together, these data suggested that FBL was a substrate of PTPN18, whereas Y313 was a major target for dephosphorylation.

### PTPN18, but not PTPN18 R451A, decreases the protein expression level of FBL by promoting its ubiquitin‒proteasome degradation

**Fig. 4:**
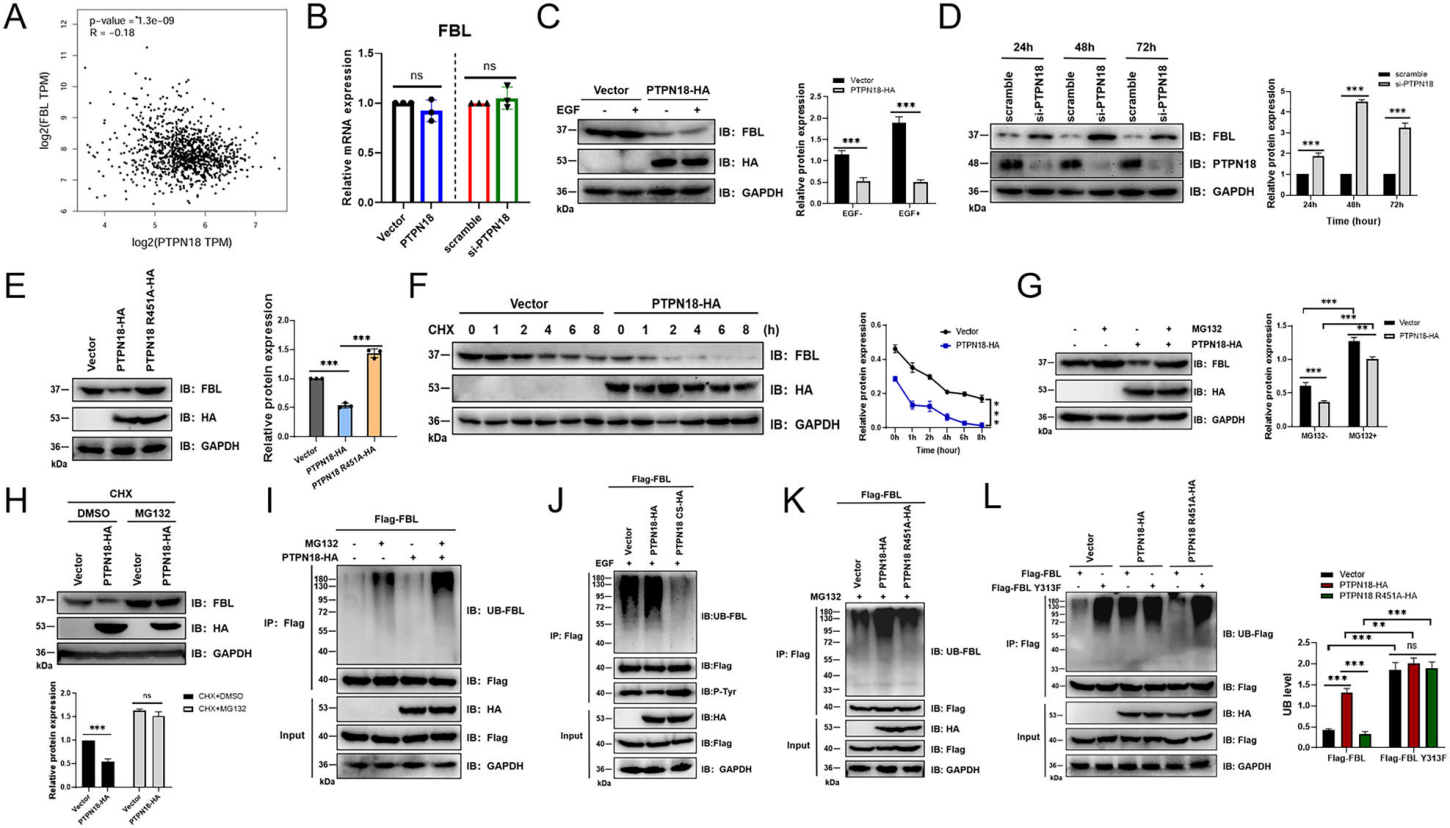
PTPN18, but not PTPN18 R451A, decreases the protein expression level of FBL by promoting its ubiquitin proteasome degradation. **A** The GEPIA database was used to analyze the association between PTPN18 and FBL. **B** QRT‒PCR analysis of the effect of PTPN18 on FBL mRNA levels. **C** Effect of PTPN18 on FBL protein expression in MCF7 cells in the presence or absence of EGF stimulation. **D** PTPN18 deletion enhances FBL protein expression. The right-hand side shows the FBL protein level statistics in a bar graph. **E** Effect of PTPN18 R451A on FBL protein expression levels. **F** Effect of PTPN18 on FBL protein levels after treatment with different concentrations of CHX (10 μg/ml), a protein synthesis inhibitor. Right: FBL protein level change line plot. **G** MG132 (10 μM, 6 h) inhibits the degradation of FBL caused by the overexpression of PTPN18. The right panel shows a protein statistical bar chart. **H** Compared with the control, the combined use of CHX and MG132 nearly abolishes the regulation of FBL protein expression by PTPN18. **I** Overexpression of PTPN18 promotes the ubiquitination of FBL. **J** The PTPN18 CS reduces the ubiquitination of FBL. **K** PTPN18 R451A reduces the level of ubiquitination of FBL. **L** Compared with the FBL WT, the FBL Y313F mutation increases the level of ubiquitination of FBL. All the experimental data were verified in at least three independent experiments (*n* ≥ 3). ns, not significant; **P < 0.01; ***P < 0.001.

The results of gene correlation analysis in the GEPIA database revealed a significant negative correlation between PTPN18 and FBL (Fig. 4A). The results of the expression level analysis revealed that PTPN18 affected mainly the expression of FBL at the protein level, and this effect required the combination of the two (Fig. 4B-E). Treatment with the protein synthesis inhibitor cycloheximide (CHX) at different time points revealed that PTPN18 overexpression promoted the degradation of the FBL protein (Fig. 4F), while knockdown inhibited its degradation (Fig. S3A). PTPN18 promotes degradation of the substrate HER2 via the proteasome pathway [9], and FBL can be degraded by the proteasome [24, 25]. The application of the proteasome inhibitor MG132 significantly increased endogenous FBL expression levels (Fig. S3B). The addition of MG132 inhibited the degradation of FBL induced by PTPN18 overexpression (Fig. 4G) and enhanced the inhibitory effect of PTPN18 knockdown (Fig. S3C). Compared with the control treatment, the combined treatment with CHX and MG132 nearly abolished the effect of PTPN18 on FBL protein levels (Fig. 4H, S3D). In addition, overexpression of PTPN18 increased the ubiquitination level of FBL (Fig. 4I, S3E), indicating that PTPN18 could promote its proteasomal degradation by increasing FBL ubiquitination, which led to a decrease in its expression level. However, PTPN18 CS and PTPN18 R451A reduced FBL ubiquitination (Fig. 4J–L), suggesting that the ubiquitination of FBL by PTPN18 required the phosphatase activity of PTPN18 and its binding. Compared with wild-type FBL, the FBL Y313F mutation enhanced FBL ubiquitination (Fig. 4L), suggesting that the phosphorylation state could influence ubiquitination.

### PTPN18 inhibits its downstream function by negatively regulating FBL, whereas PTPN18 R451A fails

**Fig. 5:**
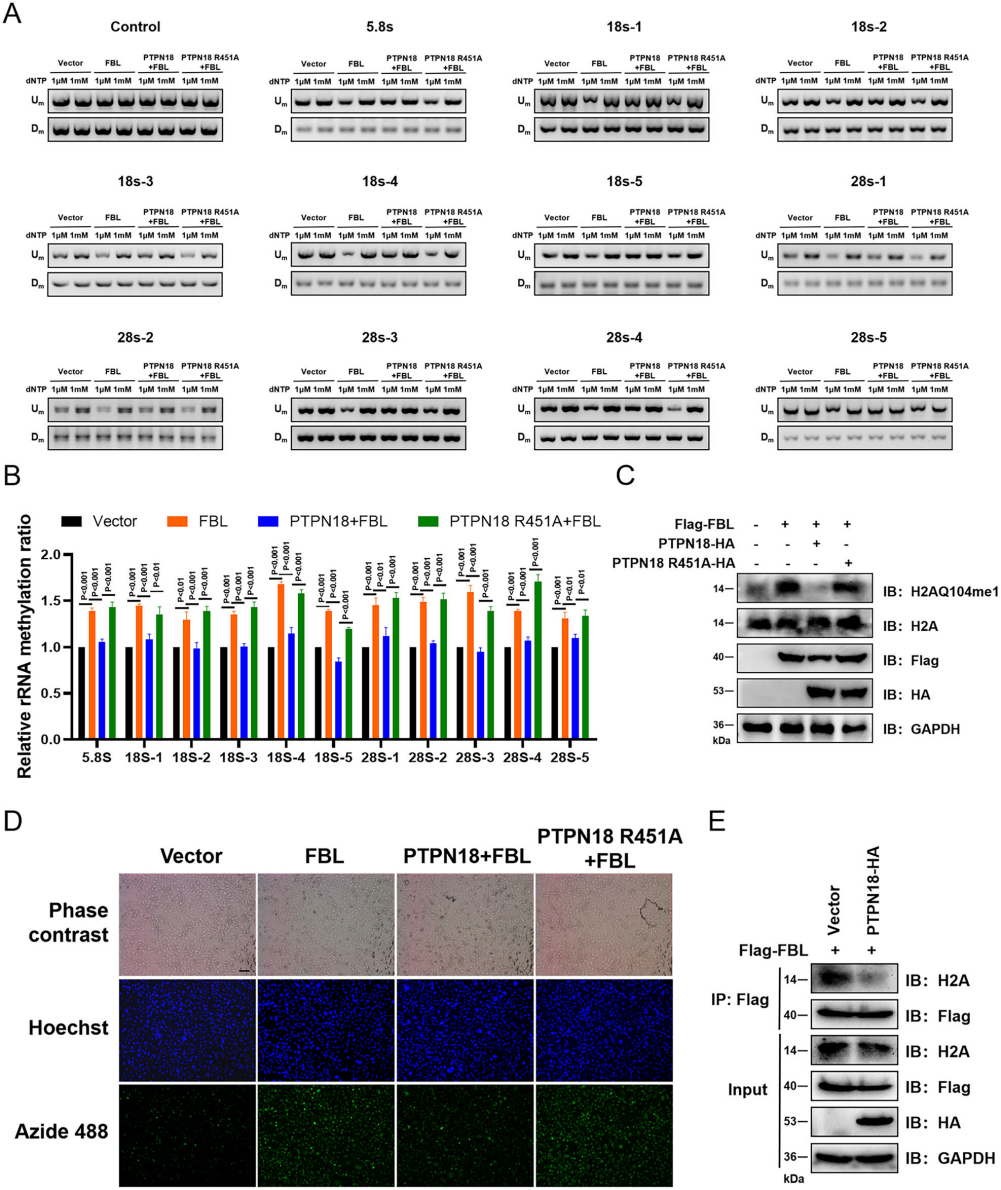
Regulation of the downstream functions of FBL by PTPN18 requires a combination of both. **A** The 2′-O-Me levels in rRNA were measured by RTL-P. Total RNA was reverse transcribed (RT) with RT primers at low (1 μM) or high (1 mM) dNTP concentrations. The obtained cDNAs were then amplified with primer pairs corresponding to upstream (Um) or downstream (Dm) regions of specific methylation sites. **B** Densitometric analysis of the data from A is shown as the signal intensity ratio of PCR products at low dNTP concentrations (1 μM) to those at high dNTP concentrations (1 mM). Methylation levels in vector cells were set close to 1. **C** PTPN18 and FBL regulate histone H2AQ104me1 levels. **D** Click chemistry-based EU-488 RNA synthesis assay for fluorescent labeling and detection of newly synthesized RNA in MDA-MB-231 cells. Scale bar, 100 μm. **E** Co-IP assays revealed the effect of PTPN18 on the binding of FBL to histone H2A. All the experimental data were verified in at least three independent experiments (*n* ≥ 3).

FBL can catalyze the methylation of rRNA 2′-O and H2AQ104, guide the cleavage and processing of pre-rRNA, and promote rRNA transcription [18, 26–28]. The effect of PTPN18 on rRNA 2′-O methylation was subsequently investigated by reverse transcription at a low deoxyribonucleoside triphosphate (dNTP) concentration followed by PCR (RTL-P) [26, 29, 30]. The overexpression of FBL resulted in significantly elevated levels of 2′-O-Me in all the rRNA fragments detected, and the overexpression of PTPN18, but not PTPN18 R451A, decreased the methylation levels of rRNA, indicating that PTPN18 regulated rRNA 2′-O-Me by interacting with FBL (Fig. 5A, B; S4A, B). In addition, PTPN18 reversed the effects of FBL on H2AQ104 methylation and RNA synthesis, whereas PTPN18 R451A did not, demonstrating that the regulation of FBL downstream function by PTPN18 required maintenance of the interaction between the two (Fig. 5C, D; S4C, D). Finally, PTPN18 overexpression weakened the interaction between FBL and H2A, suggesting that PTPN18 might competitively bind FBL with H2A (Fig. 5E, S4E).

### PTPN18 can regulate the proliferation and apoptosis of breast cancer cells by interacting with FBL

**Fig. 6:**
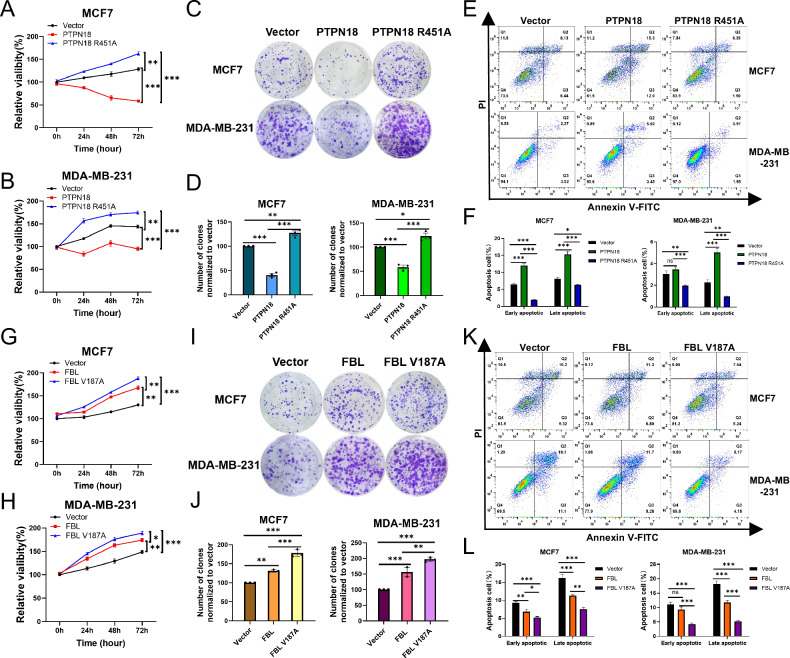
PTPN18 regulates the proliferation and apoptosis of breast cancer cells by interacting with FBL. **A**−**H** The viability of MCF7 (**A**, **G**) and MDA-MB-231 (**B**, **H**) cells was measured using a CCK8 assay at various time points. The data were normalized against the vector groups. **C**, **I** A colony formation assay was used to determine the long-term effects of the PTPN18 interaction with or without FBL on breast cancer cell growth in vitro. **D**, **J** ImageJ was used to count the number of colonies formed in Panels C and I and perform relative quantification. **E**, **K** The apoptosis of MCF7 and MDA-MB-231 cells was assessed by flow cytometry analysis using Annexin V-FITC and PI staining. **F**, **L** Quantitative analysis of the flow cytometric apoptosis rates shown in Panels E and K. All the experimental data were verified by at least three independent experiments (*n* ≥ 3). *P < 0.05, **P < 0.01, ***P < 0.001, ns, not significant.

Given the close association of PTPN18 and FBL with breast cancer and their respective effects on cell proliferation and apoptosis [9, 19, 31, 32], we next explored the effects of PTPN18 interacting with FBL on breast cancer proliferation and apoptosis. The results of the CCK8 cell viability assay revealed that the PTPN18 R451A mutation reversed the inhibitory effect of PTPN18 on cell viability in both MCF7 cells and MDA-MB-231 cells (Fig. 6A, B), and the results of the colony formation assay were consistent with these findings (Fig. 6C, D). The results of flow cytometric apoptosis assays revealed that the proapoptotic effect of PTPN18 on both early and late apoptosis was significantly attenuated when the noninteracting plasmid PTPN18 R451A was overexpressed (Fig. 6E, F). Immediately afterward, we disrupted the interaction of PTPN18 with FBL by overexpressing the FBL V187A mutant plasmid and found that the viability (Fig. 6G, H), clonogenic ability (Fig. 6I, J), and ability to inhibit apoptosis (Fig. 6K, L) of breast cancer cells were further enhanced compared with those in the FBL overexpression group. The results showed that PTPN18 could regulate the proliferation and apoptosis of breast cancer cells by interacting with FBL.

### Effect of the interaction of PTPN18 with FBL on tumor formation

**Fig. 7:**
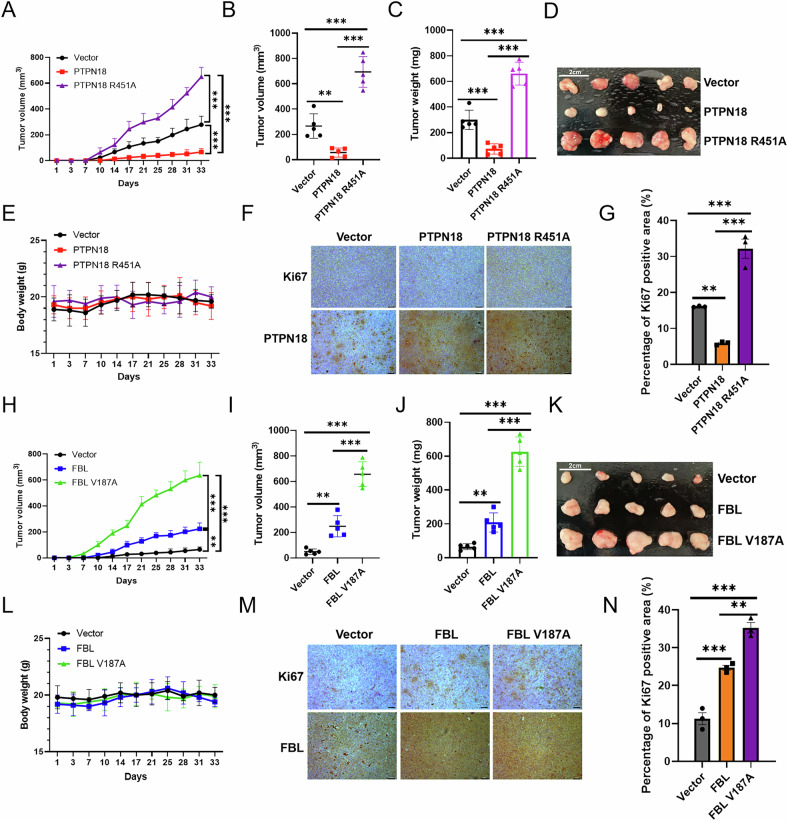
Effect of the PTPN18 interaction with FBL on tumor formation. **A**, **H** Tumor growth curve of MDA-MB-231 cells in nude mice. Tumor volumes were calculated twice per week using calipers (*n* = 5 per group). **B**, **I** Average tumor volume at the end of the experiment. **C**, **J** Statistical bar graphs of tumor weights. **D**, **K** Subcutaneous ectopic tumors in nude mice were harvested and photographed. Scale bar, 2 cm. **E**, **L** Body weight change curves of the nude mice bearing the MDA-MB-231 xenografts. **F**, **M** Ki67 expression in mouse xenografts was detected by immunohistochemistry. Scale bar, 50 μm. **G**, **N** Quantification of Ki67-positive cells within the tumor tissues is shown in a bar graph from three random field sections. All the experimental data were verified in at least three independent experiments (*n* ≥ 3). **P < 0.01; ***P < 0.001.

Next, subcutaneous tumor formation experiments in nude mice were performed to explore the effect of the interaction of PTPN18 with FBL on tumor generation at the animal level. We injected MDA-MB-231 cells overexpressing different plasmids subcutaneously into immunodeficient nude mice and measured the tumor volume (Fig. 7A, H) and body weight (Fig. 7E, L) of the mice twice a week after tumor formation. The tumors were photographed when they were ~10–15 mm in diameter (Fig. 7D, K), and the volume (Fig. 7B, I) and weight (Fig. 7C, J) of the tumors in each group were determined. The experimental results revealed that PTPN18 could inhibit tumor growth and that FBL could promote tumor growth, whereas the PTPN18 R451A mutation and FBL V187A mutation disrupted the interaction between the two, resulting in further tumor growth, which indicated that the inhibitory effect of PTPN18 on tumor growth could be achieved through the negative regulation of the interacting protein FBL. The body weights of the mice did not differ significantly between the treatment groups (Fig. 7E, L). The nuclear proliferation marker Ki67 is measured in many types of cancer, including primary breast cancer [33, 34]. The expression of Ki67 in tumor tissues detected by immunohistochemistry revealed that the expression level of Ki67 increased when PTPN18 did not interact with FBL (Fig. 7F, G, M, N). The above experimental results verified that PTPN18 inhibited the growth of breast cancer by interacting with FBL at the animal level.

### PTPN18 can regulate the proliferation and apoptosis of breast cancer cells through the dephosphorylation of FBL Y313

Given the regulatory effect of PTPN18 on FBL tyrosine phosphorylation levels, we next explored the effects of PTPN18 enzymatic activity on breast cancer cell proliferation and apoptosis. PTPN18 CS resulted in increased cell proliferation (Fig. S5A, B) and colony formation ability (Fig. S5C, D) and attenuated proapoptotic effects (Fig. S5E, F), indicating that the regulatory effect of PTPN18 on breast cancer cell proliferation and apoptosis required the involvement of its activity. Overexpression of FBL increased the proliferative activity and clonogenic ability of breast cancer cells (Fig. S5G-J) and inhibited apoptosis (Fig. S5K, L). However, this cancer-promoting effect of FBL was reversed when the FBL Y313 site regulated by PTPN18 was mutated, suggesting that FBL exerted cancer-promoting effects through the Y313 site, whereas PTPN18 could regulate breast cancer cell proliferation and apoptosis through dephosphorylation at this site (Fig. S5G-L, S6A-E).

### Effect of PTPN18 enzyme activity on tumor formation

We further explored the effect of PTPN18 enzymatic activity on tumor generation in vivo. The results of dynamic volume growth curves (Fig. S7A, H) and final volume (Fig. S7B, I) and weight (Fig. S7C, J) statistics and immunohistochemistry (Fig. S7F, G, M, N) analyses of tumors showed that PTPN18 CS resulted in increased tumor volume, weight, and Ki67 positivity rates compared with PTPN18 wild-type in the absence of significant differences in mouse body weight (Fig. S7E, L), suggesting that enzyme activity was essential for PTPN18 to play a tumor suppressor role in breast cancer. FBL overexpression promoted tumor growth and Ki67 expression, whereas the FBL Y313F mutation inhibited tumor growth and Ki67 expression, which indicated that the Y313 site was essential for FBL to promote breast cancer growth, and it was speculated that PTPN18 played a tumor suppressor role through dephosphorylation of this site. Panels S7D and K show photographic images of tumors ultimately harvested subcutaneously from nude mice. The above experimental results were generally consistent with those at the cellular level.

### PTPN18 regulates MAPK signaling through FBL and subsequently affects the proliferation and apoptosis of breast cancer cells

**Fig. 8:**
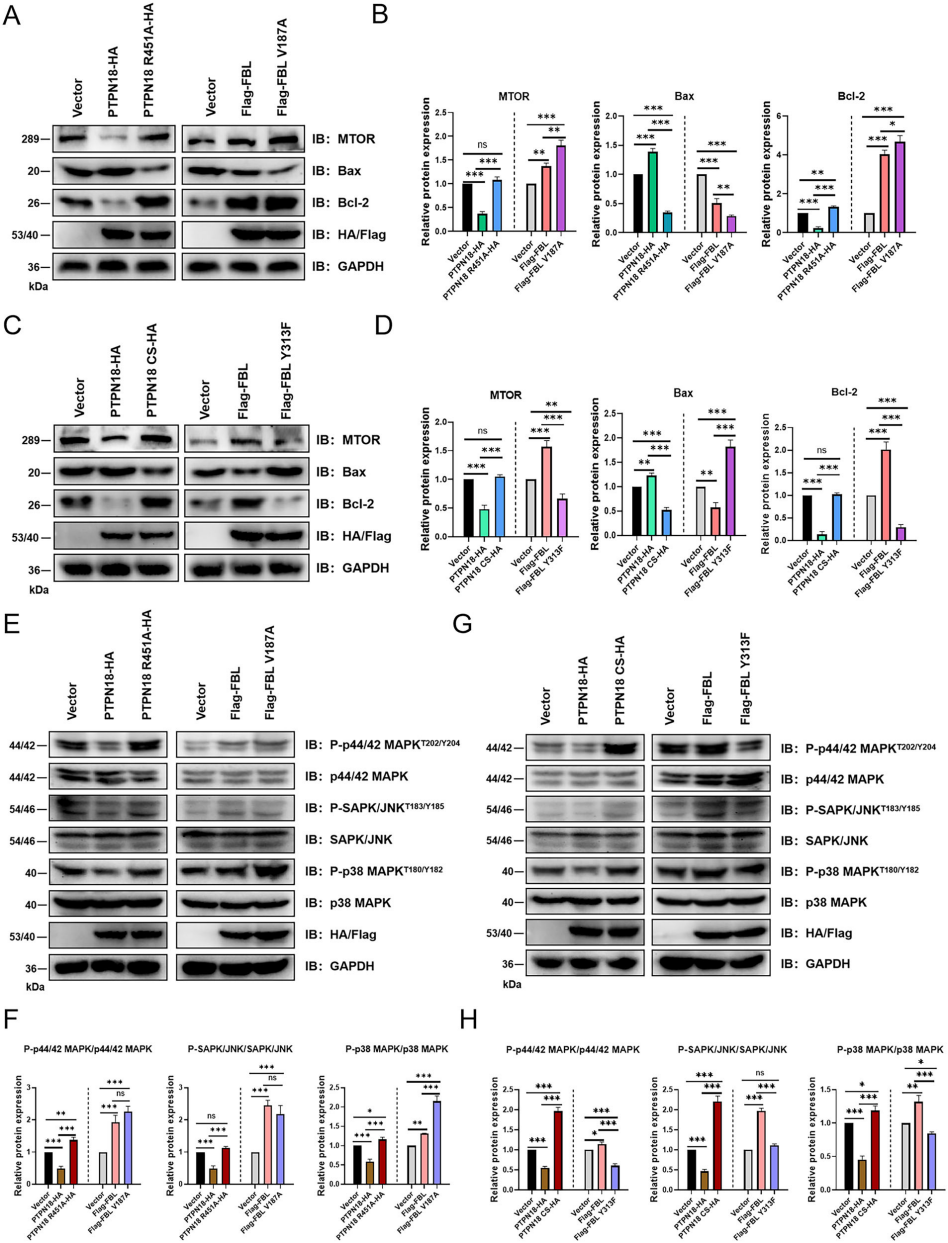
PTPN18 affects the expression of key proteins involved in proliferation and apoptosis and regulates MAPK signaling through FBL. **A** Effect of PTPN18 interaction with FBL on the expression of MTOR, Bcl-2, and Bax. **C** Effect of PTPN18 CS and FBL Y313F on MTOR, Bcl-2, and Bax expression levels in response to EGF stimulation. **B**, **D** Panels (**B** and **D**) show the statistical data for MTOR, Bcl-2, and Bax protein expression from (**A** and **C**) respectively. **E** Effect of the PTPN18 interaction with FBL on the phosphorylation levels of the key MAPK signaling pathway proteins p44/42 MAPK, SAPK/JNK and p38 MAPK. MCF7 cells were stimulated with 100 ng/mL EGF for 30 min. **G** PTPN18 CS and FBL Y313F regulate the phosphorylation levels of p44/42 MAPK, SAPK/JNK, and p38 MAPK. EGF was applied at a concentration of 100 ng/ml for 30 min. **F**, **H** Panels (**F**, **H**) show the statistics for the phosphorylation levels of p44/42 MAPK, SAPK/JNK, and p38 MAPK from E and G, respectively. ns, not significant, *P < 0.05, **P < 0.01, and ***P < 0.001 from three independent experiments (*n* = 3).

Mammalian target of rapamycin (mTOR) is a Ser/Thr protein kinase that is a central regulator of cellular growth and survival [35, 36]. The protein expression of Bcl-2 suppresses apoptosis, whereas that of BAX promotes apoptosis [37, 38]. Overexpression of PTPN18 R451A or FBL V187A resulted in further increases in the expression levels of MTOR and Bcl-2 and decreases in the expression level of Bax (Fig. 8A, B). Compared with those in the PTPN18 experimental group, the expression of MTOR and Bcl-2 increased and that of Bax decreased in the PTPN18 CS group. However, compared with the FBL group, the FBL Y313F group showed the opposite regulatory trend (Fig. 8C, D). Consistent with the results of previous phenotypic experiments, PTPN18 regulated the expression of proteins critical for proliferation and apoptosis in breast cancer cells by regulating the Y313 site of FBL.

The KEGG pathway database [39] revealed that protein tyrosine phosphatases could regulate cell proliferation and apoptosis by dephosphorylating three key points of the MAPK signaling pathway (Fig. S8), and negative regulation of the MAPK signaling pathway by protein tyrosine phosphatases has been confirmed in the literature [40–42]. Western blot experiments revealed that PTPN18 overexpression indeed decreased the phosphorylation levels of p44/42 MAPK, SAPK/JNK, and p38 MAPK, whereas FBL overexpression increased their phosphorylation levels. However, once PTPN18 lost its interaction with FBL, the phosphorylation level of MAPK signaling pathway proteins increased, which indicated that PTPN18 could regulate the MAPK signaling pathway through its interaction with FBL (Fig. 8E, F). PTPN18 CS reversed the dephosphorylating effect of PTPN18 on p44/42 MAPK, SAPK/JNK, and p38 MAPK. The overexpressed FBL Y313F mutant group attenuated MAPK pathway activation by FBL, suggesting that the Y313 site of FBL was essential for activation of the MAPK signaling pathway, whereas dephosphorylation of this site by PTPN18 negatively regulated the MAPK signaling pathway (Fig. 8G, H). The above results showed that PTPN18 could negatively regulate the MAPK signaling pathway by binding to FBL and dephosphorylating Y313, thereby regulating the proliferation and apoptosis of breast cancer cells.

## Discussion

PTPN18 is closely related to the development of breast cancer [8–11], but its mechanism of action in breast cancer remains largely unknown. In this study, a novel protein, FBL, that interacts with PTPN18 was identified, and the key amino acid sites R451 (PTPN18) and V187 (FBL) controlling the interaction between the two, as well as the specific site Y313 where PTPN18 dephosphorylates FBL, were identified. The discovery and study of these amino acid sites with special functions not only help elucidate the functional regulatory mechanism of proteins in cells but also improve the precision and safety of drugs, accelerate the process of drug research and development, reduce costs, and provide strong support for personalized medicine and precision medicine [43–45].

The results of gene correlation analysis in the GEPIA database revealed that PTPN18 expression was significantly negatively correlated with FBL. In addition to the above validated regulation of FBL dephosphorylation by PTPN18, we further investigated the effect of PTPN18 on FBL expression. The results showed that PTPN18, but not PTPN18 R451A, could downregulate the protein expression level of FBL by promoting its ubiquitin‒proteasome degradation. In addition, the regulation of FBL ubiquitination by PTPN18 required the maintenance of enzymatic activity, and compared with wild-type FBL, the FBL Y313F mutant, which cannot be phosphorylated, had a higher level of ubiquitination. This finding indicated that the phosphorylation status of FBL affected its ubiquitination level.

In view of the negative regulation of FBL phosphorylation and protein expression by PTPN18, this study further investigated the effect of PTPN18 on the downstream function of FBL. The experimental results revealed that PTPN18 could indeed regulate the methylation of rRNA 2′-O and histone H2AQ104 as well as RNA synthesis through FBL, whereas the PTPN18 R451A mutation could not. In addition, PTPN18 overexpression reduced H2A binding to FBL, suggesting that PTPN18 might have a competitive binding relationship with H2A to FBL. This competition may have broad effects on a variety of biological processes within cells, including nucleolar function, chromatin structure, cell cycle regulation, protein stability, and signaling [9–19, 28, 46].

Given that both PTPN18 and FBL can regulate the occurrence and development of breast cancer [9–11, 19], we next explored the effects of the interaction between PTPN18 and FBL on breast cancer cell survival and apoptosis. The results at the cellular and animal levels confirmed that PTPN18 indeed inhibited breast cancer cell proliferation, promoted apoptosis, and thereby inhibited tumor growth in an FBL-dependent manner. In addition, western blot results revealed that PTPN18 could regulate MAPK signaling and the expression of key downstream proliferation and apoptosis proteins by interacting with FBL to function as a tumor suppressor. PTPN18 can promote the development of colorectal and endometrial cancer [47, 48], and these functional differences may be the result of a combination of interacting protein target remodeling, dynamic regulation of subcellular localization, and the tumor microenvironment.

**Fig. 9:**
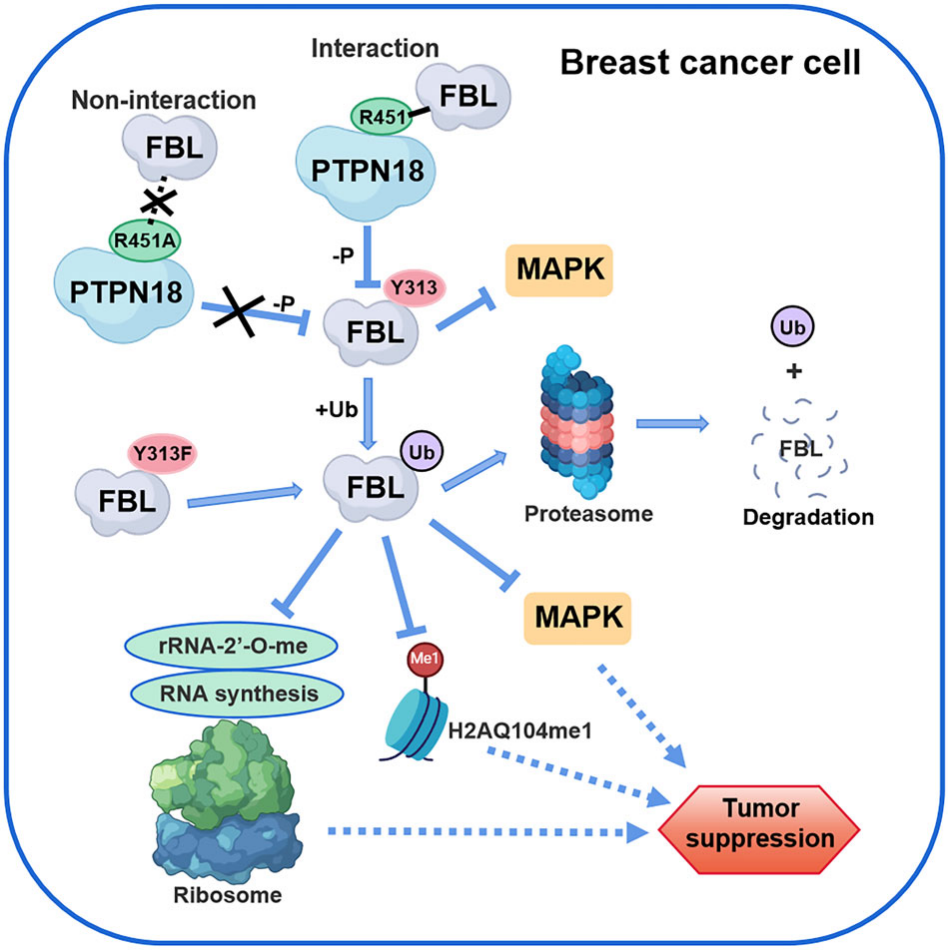
Schematic representation of PTPN18 exerting tumor suppressive effects on breast cancer through the negative regulatory protein FBL at the R451 site. In the normal state, wild-type PTPN18 can interact with FBL through its own R451 site and dephosphorylate the FBL Y313 site. The dephosphorylation state of FBL enhances its own ubiquitination and promotes its proteasomal degradation, resulting in reduced levels of FBL expression. FBL inhibited by PTPN18 together with decreased expression attenuates the promoting effect on the MAPK signaling pathway, RNA synthesis, and methylation of rRNA 2’-O and histone H2AQ104, ultimately leading to the inhibition of breast cancer development. However, when the R451 site of PTPN18 is mutated to A, PTPN18 cannot interact with FBL and loses the pathway that depends on FBL to exert its tumor suppressor effect. The dephosphorylation state of FBL Y313F promotes ubiquitinated proteasomal degradation of FBL.

In summary, this study revealed and dissected a key mechanistic pathway through which FBL-dependent PTPN18 regulates cellular processes involved in breast carcinogenesis. The R451 site was found to be critical for the tumor suppressive effects of PTPN18 in breast cancer because it regulates the phosphorylation and ubiquitination of FBL, affecting downstream methylation as well as MAPK signaling pathways (Fig. 9). PTPN18 negatively regulates FBL; blocks its rRNA processing, ribosome assembly and other functions; inhibits protein synthesis in tumor cells; limits proliferation; promotes apoptosis; and has FBL-dependent tumor suppressor effects. When FBL is absent, the subcellular localization of PTPN18 is altered to reduce FBL dependence and enhance its substrate effects on HER2 and ETS1, among others [9, 10], and FBL independently inhibits breast cancer progression. PTPN18 tumor suppression requires multisubstrate cooperation, and alternative mechanisms ensure the adaptability of life to external changes, which is in line with its evolutionary nature. In this study, we showed that PTPN18 could synergistically regulate cellular processes through a variety of different protein posttranslational modifications and then flexibly regulate the physiological function of cells. The dissection of the PTPN18 protein interaction network is extremely important for understanding cell biology, driving drug development, and revealing disease mechanisms, especially in breast cancer.

## Materials and methods

### Cell culture and Transfection

The HEK293, MCF7, and MDA-MB-231 cell lines were obtained from the Chinese Academy of Sciences (Shanghai, China) and maintained in DMEM (HyClone, Waltham, MA, USA) supplemented with 10% fetal bovine serum and 1% penicillin/streptomycin (P/S) at 37 °C in a humidified incubator with 5% CO_2_. In accordance with the instructions, interference with gene expression levels was achieved by using transfection reagent (Neofect, Beijing, China). For transfection with knockdown plasmids, transfection was performed again 24 h after normal transfection to obtain better knockdown efficiency. The sequences of the siRNAs used in this study are shown in Supplementary Table 1.

### Protein extraction and western blotting

Total cell lysates were harvested with precooled cell lysis buffer supplemented with 1% protease inhibitor (Selleck, Houston, USA) and phosphatase inhibitor cocktail (Selleck, Houston, USA). A BCA kit was used to assess the total protein concentration of the supernatant. Equal amounts of extracted proteins were loaded and subjected to SDS‒polyacrylamide gel electrophoresis. Following electrophoresis, the proteins were cryogenically transferred to polyvinylidene fluoride (PVDF) membranes via the sandwich method at a constant voltage of 100 V. The membranes were blocked with 5% BSA for 1 h at room temperature and then incubated overnight at 4 °C with diluted primary antibody. The following day, the primary antibodies were removed by washing with Tris-buffered saline (TBS) containing 0.1% Tween-20 and incubated with a secondary antibody for 1–2 h at room temperature with gentle shaking. Finally, the membranes were scanned, visualized, and quantitatively analyzed using a ChemiDoc XRS + Gel Imaging Analysis System (Bio-Rad, Hercules, CA, USA) and Image Lab software. Antibody information is listed in Supplementary Table 2. The original western blot map is presented in the Supplementary Material “Original Western Data”.

### Coimmunoprecipitation (Co-IP) assay

After being subjected to different treatments, the cells were lysed in cold RIPA lysis buffer supplemented with protease inhibitor cocktail and phosphatase inhibitor cocktail. The cell lysates were centrifuged at 12,000 × g and 4 °C for 15 min to remove cell debris and incubated with the appropriate primary antibodies overnight at 4 °C by inversion. The following day, the conjugates were incubated with protein A/G agarose beads (Beyotime, Shanghai, China) for 2 h at 4 °C. The beads were washed five times with cold lysis buffer, and the supernatant was discarded and boiled with 1 × SDS loading buffer for 10 min for subsequent western blot analysis.

### Co-IP and MS

First, the empty vector and PTPN18 plasmid were overexpressed in HEK293 cells with good growth status, and the cells were collected 48 h later to obtain IP samples according to the Co-IP experimental procedures described above. Subsequently, some samples were collected to ensure that PTPN18 had been well precipitated by the western blot experiments. IP samples with the PTPN18 pellet and its interacting proteins were appropriately resolved by polyacrylamide gel electrophoresis followed by Coomassie blue staining. After elution to the position where the protein band could be seen, the strips in the protein band were cut and identified by mass spectrometry (Jingjie PTM Biolabs, Inc., Hangzhou, China) to obtain proteins that might interact with PTPN18. The differentially expressed protein obtained was subsequently identified as a candidate protein for PTPN18 interaction by excluding nonspecific binding compared with controls. GO/KEGG enrichment analysis of these candidate proteins was performed using the SRplot platform (https://www.bioinformatics.com.cn/basic_local_go_pathway_enrichment_analysis_122) [49]. The GEPIA2 database was used to analyze the association of PTPN18 with candidate proteins in breast cancer (http://gepia2.cancer-pku.cn/#correlation) [50].

### Immunofluorescence colocalization assay

HEK293 cells were cultured and placed on dedicated coverslips in 24-well plates. The cells were fixed using 4% paraformaldehyde for 30 min at room temperature and then permeabilized with PBS containing 0.3% Triton X-100 for 30 min at room temperature. After blocking with 3% BSA (prepared in PBS for dissolution) for 1 h at room temperature, the cells were incubated overnight at 4 °C with primary antibodies. The following day, the primary antibody was removed by washing with PBST (0.1% Tween) and incubated with the secondary antibody for 1 h at room temperature in the dark. DAPI (Absin, Shanghai, China) was used to stain the nuclei. The coverslips were removed and inverted onto clean slides dropwise with an appropriate amount of anti-fluorescence quenching mounting solution (Beyotime, Shanghai, China) and observed under a fluorescence microscope (Leica Microsystems, Wetzlar, Germany).

### Proximity Ligation Assay

Well-grown adherent MCF7 cells were gently rinsed three times with prechilled PBS for 1 min each to remove medium residues. Afterward, 4% paraformaldehyde was added and fixed at room temperature for 20 min; 0.3% Triton X-100 was added after 3 PBS rinses followed by permeabilization at room temperature for 30 min. After permeabilization, the cells were rinsed 3 times with PBS and blocked at 37 °C for 30 min, and after blocking, a proximity ligation assay was performed using a Duolink In Situ PLA Kit (red) (Sigma‒Aldrich, St. Louis, USA) according to the Duolink® PLA Fluorescence Protocol. Images were acquired under a fluorescence microscope. If the distance between the two proteins is < 40 nm (i.e., interaction occurs), the probe can be connected and initiate rolling circle amplification, and finally, the presence and location of the interaction are visually displayed by the fluorescence signal. No fluorescence signal was detected in the control group with or without only one protein primary antibody.

### CCK8 cell proliferation viability assay

Appropriate amounts of treated cells were seeded in 96-well plates containing 100 μL of culture medium according to the growth rate of different cells. After the cells were completely attached, the medium was replaced with fresh medium, 10 μL of CCK8 reagent (Absin, Shanghai, China) was added, and the cells were incubated for 2 h. The absorbance was read at 450 nm at 0 h using an iMark full-function microplate reader (Bio-Rad, Hercules, CA, USA). The OD values were subsequently recorded after 24 h, 48 h and 72 h of culture. Cell proliferation was calculated from the results of the CCK8 assay. Five replicate wells were established for each treatment group, and all tests were independently repeated three times.

### Colony formation assay

Treated breast cancer cells were seeded in 6-well plates at a density of ~2000 cells/well. The media was changed twice weekly according to the cell growth status. After ~2 weeks of culture, the cells were fixed with 4% paraformaldehyde and stained with crystal violet (Beyotime, Shanghai, China). Finally, the excess dye was removed by washing with water, and the sample was air-dried at room temperature. Colony formation was assessed by counting the number of colonies on each plate with ImageJ software. All tests were independently repeated three times.

### Flow cytometric apoptosis detection

The apoptosis of breast cancer cells was detected by flow cytometry. The cells (1 ~ 2.5 × 10^6^) were washed and collected with pre-chilled PBS. They were then resuspended by the addition of 100 μL of 1 × binding buffer, after which 5 μL of annexin V-FITC and 5 μL of PI staining solution (BD, Franklin Lakes, USA) were added with gentle mixing for 10 min at room temperature in the dark. A control group without annexin V-FITC/PI was also set up for finding cell clusters and a single staining group stained only with PI or FITC for subsequent adjustment compensation. After 400 μL of 1 × binding buffer was added and mixed, the samples were filtered with gauze and detected by flow cytometry within 1 h. The data were analyzed and processed with FLOWJO software.

### Quantitative real-time polymerase chain reaction (qRT‒PCR)

Total cellular RNA was extracted using TRIzol, and cDNA synthesis was performed using an All-in-One cDNA Synthesis Super Mix Kit (Bimake, Houston, USA) following the instructions for use. A total reaction volume of 20 μL per well was prepared using the 2xSYBR Green qPCR Master Mix kit (Bimake, Houston, USA), and 3–5 replicate wells were established per group. Mean cycle threshold (Ct) values were obtained for each reaction, and the relative expression was quantified using the 2^(−ΔΔC(T))^ method. The GAPDH level was used as the internal control. The sequences of the primers used for qRT‒PCR are shown in Supplementary Table 3.

### Subcutaneous xenograft tumor model

The mice used for the animal experiments were BALB/c nude mice (4–6 weeks old, female) from Beijing SPF Laboratory Animal Co., Ltd. Xenograft models were established by subcutaneous injection of control or genetically manipulated MDA-MB-231 cells in 150 μL of cell suspension (~5 × 10^6^ cells). The body weights and tumor volumes of the mice were measured twice weekly. The mice were euthanized ~30 days after the tumors grew to 10–15 mm in diameter. The tumor volume was calculated according to the following formula: volume (mm^3^) = (length × width^2^)/2. All mice were housed and handled following protocols approved by the Animal Care and Use Committee of Northeastern University. Batch number: NEU-EC-2025A024S.

### Immunohistochemical analysis

Fresh tumor tissues were fixed by immersion in 4% paraformaldehyde, dehydrated, embedded in paraffin, and sectioned at a thickness of 4 mm. In accordance with the instructions, immunohistochemical experiments were performed using a ready-to-use immunohistochemical high-sensitivity UltraSensitive^TM^ SP kit (MXB Biotechnologies, Fujian, China) and a DAB chromogenic kit (ZSGB-BIO, Beijing, China). The sections were then observed under an inverted microscope, and ImageJ was used to determine the percentage of positive cells.

### Determination of rRNA methylation level by the Reverse Transcription at Low dNTP concentrations followed by PCR (RTL-P) method

The 2′-O-methylated nucleotides can prevent reverse transcription (RT) reactions at low dNTP concentrations. Thus, RT reactions with low dNTP concentrations should yield fewer cDNA products than RT reactions with high dNTP concentrations. PCR amplification was subsequently performed with different PCR primer pairs targeting either upstream (Um) or downstream (Dm) specific methylation sites. At low dNTP concentrations, the number of Um primer PCR products decreased with increasing RNA 2′-O-Me levels. This amount was similar in the presence of high concentrations of dNTPs. Total RNA was extracted using a Total RNA Kit I (OMEGA Bio-Tek, Norcross, USA), and RT was performed using specific reverse primers targeting rRNA sequences upstream of one or more methylation sites in the presence of low (1 μM) or high (1 mM) concentrations of dNTPs according to the instructions supplied with the PrimeScript ™ II 1st Strand cDNA Synthesis Kit (Takara, Japan). The optimal range of cycle numbers was carefully checked by performing PCR analysis of a series of cycles. The PCR products were then loaded equally, separated on a 2% agarose gel, stained with GelRed dye (UElandy, Jiangsu, China), and visualized by UV transmission illumination. PCR signal intensities were analyzed using Image Lab. The methylation ratio for each group was determined by the density of the PCR bands obtained using high and low dNTP concentrations. PCR primers designed for RTL-P analysis are listed in Supplementary Table 4 [26].

### RNA synthesis assay

In this paper, the BeyoClick™ EU-488 RNA Synthesis Assay Kit (Beyotime, Shanghai, China), a method based on the incorporation of the uridine analog EU (5-ethynyl-uridine) during RNA synthesis and the subsequent click reaction to label EU with Alexa Fluor 488, was used to detect newly synthesized RNA. Prewarmed EU working solution (37 °C) was added to the test culture plate proportionally to continue incubating the cells for ~2 h for EU labeling; the culture medium was removed, 1 ml of fixative was added, and the cells were fixed at room temperature for 15 min. The cells were subsequently washed and permeabilized for 10–15 min at room temperature via the addition of permeabilizing solution. The cells were washed, Click reaction solution was added, and the plate was shaken gently to ensure that the reaction mixture could cover the sample evenly and incubated for 30 min at room temperature in the dark. Following washing, the nuclei were stained by incubation with 1 × Hoechst 33342 for 10 min at room temperature in the dark. Fluorescence detection was performed by fluorescence microscopy after the excess dye solution was removed and moderate air drying was performed.

### Statistical analysis

The data are presented as the mean of at least three independent experiments, and all the data are reported as the mean ± standard deviation (SD). Data analysis was performed using the Student’s *t*-test, one-way analysis of variance, and multiple comparisons (GraphPad Prism), and differences were considered to be statistically significant when P < 0.05.

## Data and materials availability

The data used to support the findings of this study are available from the corresponding authors upon request.

## Supplementary information

Supplementary Figure Legends

Supplementary Figure 1

Supplementary Figure 2

Supplementary Figure 3

Supplementary Figure 4

Supplementary Figure 5

Supplementary Figure 6

Supplementary Figure 7

Supplementary Figure 8

Supplementary table1-4

Original Western Data

aj-checklist
