# Supplementary material for: The R451 site is critical for PTPN18 to exert tumor suppressive effects in breast cancer through the negative regulatory interacting protein fibrillarin
Source: Cell Death Dis. 2026 Jan 20;17(1):168. doi: 10.1038/s41419-025-08395-1 (PMC12876892; doi:10.1038/s41419-025-08395-1)
Supplement: Supplementary file 1 — Supplementary Figure Legends [file 41419_2025_8395_MOESM1_ESM.docx]

**Supplementary Figure 1 Analysis of the relationship between PTPN18 expression levels and overall survival in breast cancer patients in different datasets from the Breast Cancer Integrative Platform (BCIP) database.** (A) Metabric dataset. (B) TCGA-RNA-Seq dataset. (C) TCGA-Agilent dataset. (D) GSE1456-GPL96 dataset.

**Supplementary Figure 2 Selection of the optimal conditions for EGF stimulation of tyrosine phosphorylation in FBL.** (A) Selection of the optimal concentration for EGF stimulation in MCF7 cells. The right panel shows a statistical line plot of the changes in FBL tyrosine phosphorylation levels. (B) Selection of the optimal time for EGF stimulation of FBL tyrosine phosphorylation in MCF7 cells. The right side shows the statistical plot of the changes in protein levels. (C) Tyrosine sites potentially dephosphorylated by PTPN18 above the threshold (DTU Health Tech-NetPhos-3.1). (D) Statistics of the relative FBL expression shown in Figure 5B. All the experimental data were verified in at least three independent experiments (n ≥ 3).

**Supplementary Figure 3 PTPN18 can reduce the protein expression level of FBL by promoting its ubiquitin proteasome degradation.** (A) Effect of PTPN18 knockdown on FBL protein expression in response to CHX treatment. (B) MG132 inhibited FBL degradation in a time-dependent manner. Right: FBL protein level statistical line graph. (C) MG132 enhanced the inhibitory effect of PTPN18 knockdown on FBL degradation. (D) The effect of loss of PTPN18 expression on FBL protein levels when CHX was combined with MG132 was not significantly different from the control. (E) PTPN18 knockdown decreased FBL ubiquitination. All the experimental data were verified in at least three independent experiments (n ≥ 3). * P < 0.05; ** P < 0.01; ***P < 0.001; ns, not significant.

**Supplementary Figure 4 PTPN18 inhibits its downstream function by negatively regulating FBL.** (A) RTL-P assay for determining 2′-O-Me levels in rRNA. (B) Relative quantification bar plots of methylation levels for data from A. (C) Effect of PTPN18 or FBL deletion on histone H2AQ104 methylation levels. (D) Fluorescent labeling to detect newly synthesized RNA in MCF7 cells. Scale bar, 100 μm. (E) Co-IP was performed to examine the effect of PTPN18 on the binding of FBL to histone H2A. All the experimental data were verified in at least three independent experiments (n ≥ 3).

**Supplementary Figure 5 PTPN18 can regulate the proliferation and apoptosis of breast cancer cells through the dephosphorylation of FBL Y313.** (A, B, G, H) CCK8 assay for determining the viability of MCF7 (A, G) and MDA-MB-231 (B, H) cells. (C, I) Colony forming ability of breast cancer cells in a six-well plate. (D, J) Prism was used to perform relative quantitative analysis of the data in Panels C and I. (E, K) Apoptosis was assessed by flow cytometry analyses of annexin V-FITC/PI double-stained cells after treatment. (F, L) Bar statistics of apoptotic cells for Panels E and K. All experimental data were verified in at least three independent experiments (n ≥ 3). *P < 0.05, **P < 0.01, ***P < 0.001 and nonsignificant (ns).

**Supplementary Figure 6 PTPN18 dephosphorylates FBL Y313 to inhibit breast cancer progression.** (A) A CCK8 assay was used to evaluate the proliferation and viability of the MCF7 cells at different timepoints. (B) The colony formation ability of the MCF7 cells was tested using six-well plates. (C) Prism plots were constructed for the relative quantitative analysis shown in Panel B. (D) Flow cytometry analysis was performed to assess apoptosis in MCF7 cells. (E) Panel D bar graph statistics for apoptotic cells. All experimental data were verified in at least three independent experiments (n ≥ 3). **P < 0.01, ***P < 0.001.

**Supplementary Figure 7 Effect of PTPN18 enzyme activity on tumor formation.** (A, H) Growth curves of tumors in nude mice treated with different treatments. (B, I) Statistical plots of tumor volume according to treatment group. (C, J) Statistical bar graphs of tumor weights. (D, K) Photographs of xenograft tumors. Scale bar, 2 cm. (E, L) Body weights of nude mice bearing MDA-MB-231 xenografts. (F, M) Ki67 was evaluated via IHC in mouse xenografts. Scale bar, 50 μm. (G, N) The Ki67-positive area ratio determined by ImageJ. All experimental data were verified in at least three independent experiments (n ≥ 3). *P < 0.05; **P < 0.01; ***P < 0.001.

**Supplementary Figure 8 The KEGG signaling pathway database demonstrates the important role of protein tyrosine phosphatases (PTPs) in negatively regulating key nodes of MAPK signaling pathways.**

**Supplementary Table 1. Sequences of siRNAs used in this study.**

**Supplementary Table 2. Antibodies used in this study.**

**Supplementary Table 3. Primer sequences used in this study for qRT-PCR.**

**Supplementary Table 4. Primer sequences used in this study for RTL-P.**
