# Supplementary figures and images for: The R451 site is critical for PTPN18 to exert tumor suppressive effects in breast cancer through the negative regulatory interacting protein fibrillarin
Source: Cell Death Dis. 2026 Jan 20;17(1):168. doi: 10.1038/s41419-025-08395-1 (PMC12876892; doi:10.1038/s41419-025-08395-1)

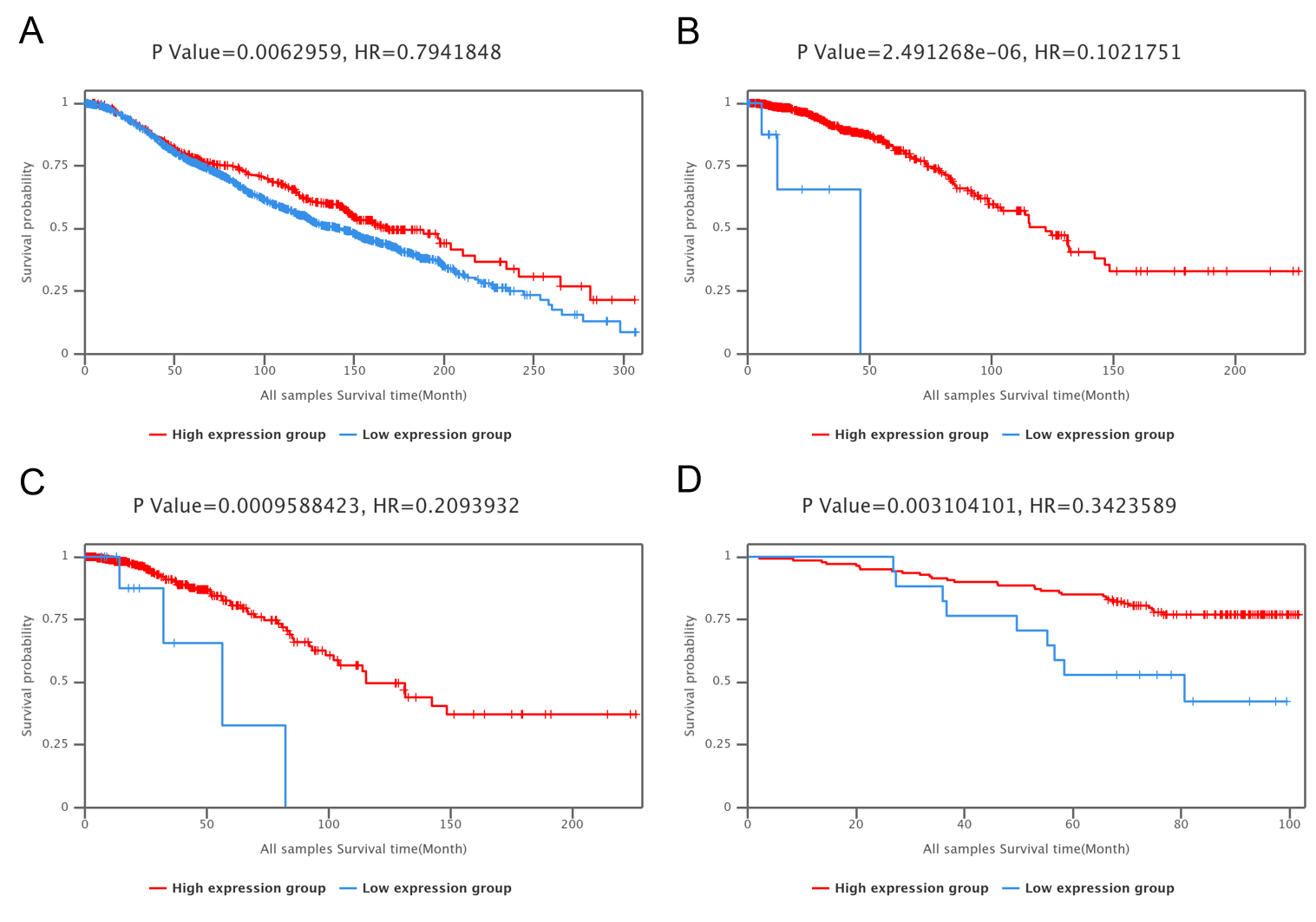

Supplement: Supplementary file 2 — Supplementary Figure 1 [file 41419_2025_8395_MOESM2_ESM.tif]

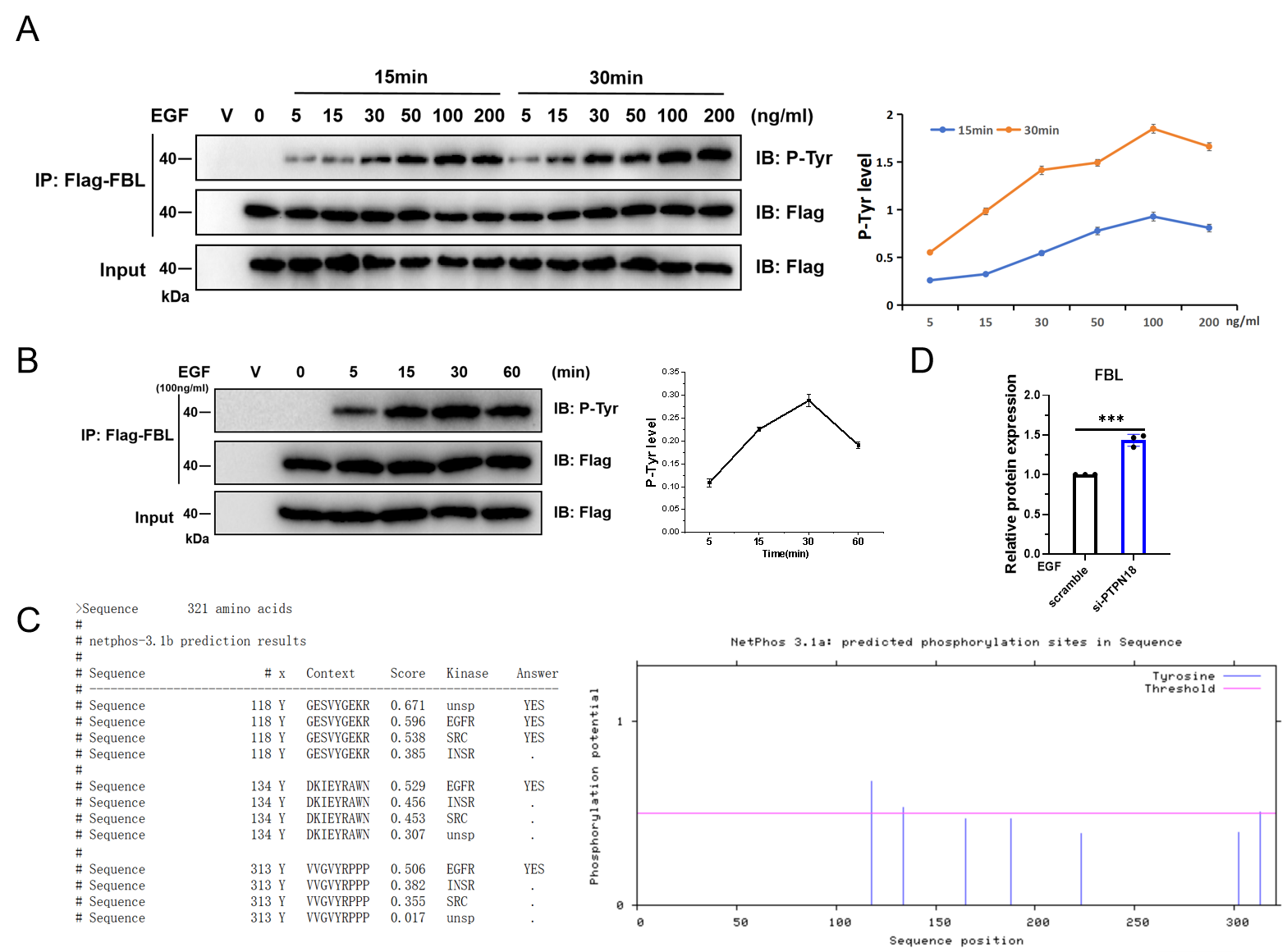

Supplement: Supplementary file 3 — Supplementary Figure 2 [file 41419_2025_8395_MOESM3_ESM.tif]

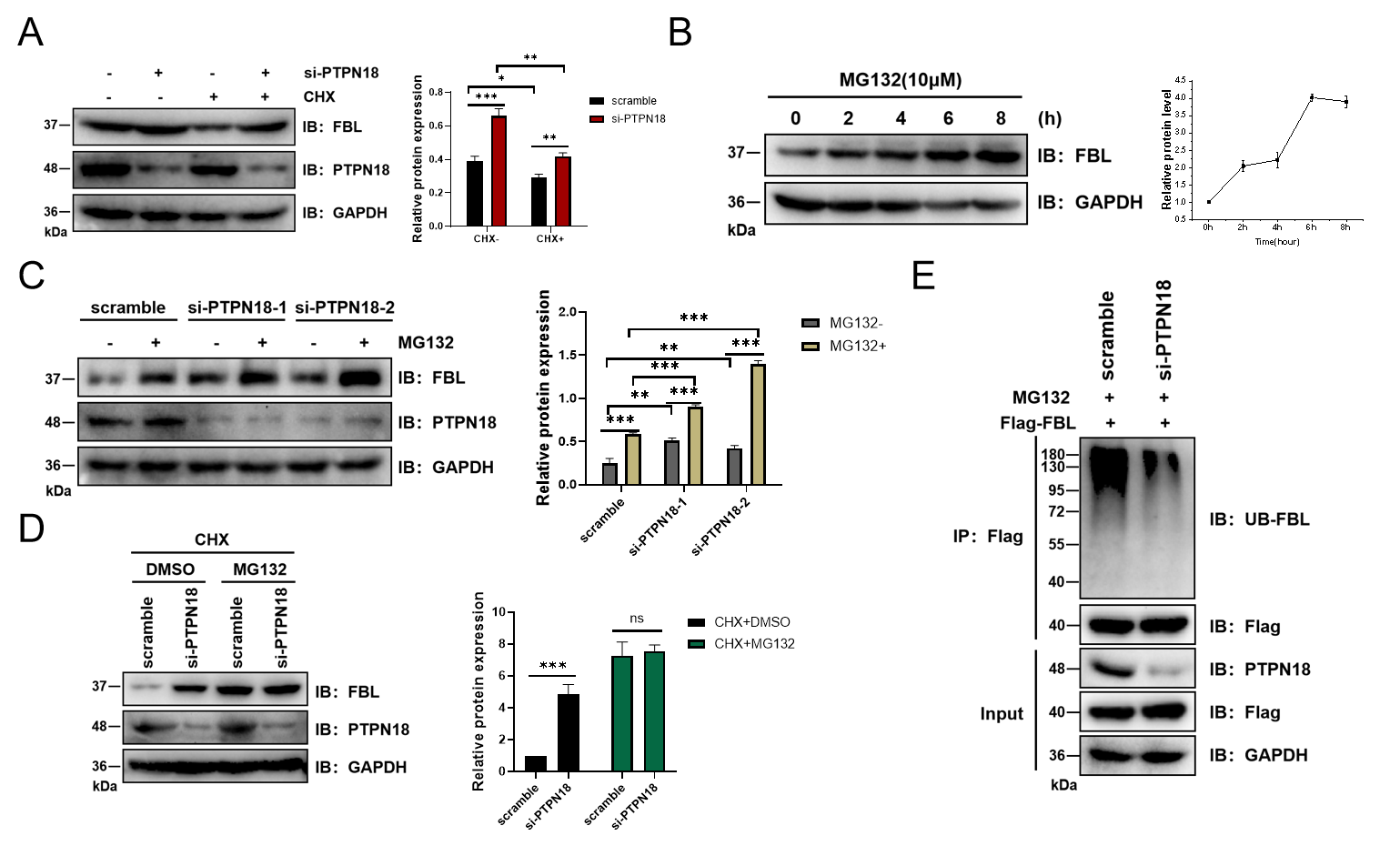

Supplement: Supplementary file 4 — Supplementary Figure 3 [file 41419_2025_8395_MOESM4_ESM.tif]

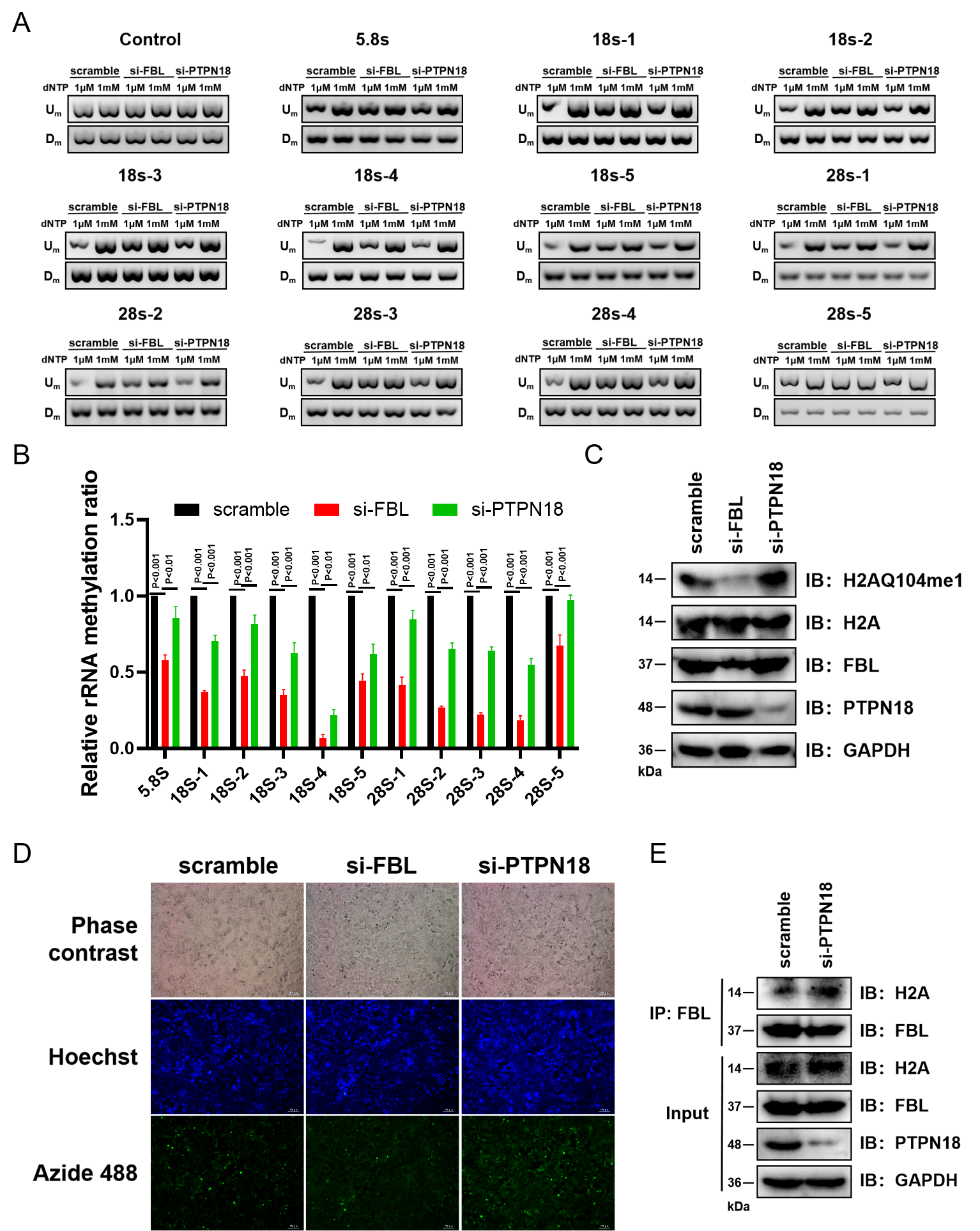

Supplement: Supplementary file 5 — Supplementary Figure 4 [file 41419_2025_8395_MOESM5_ESM.tif]

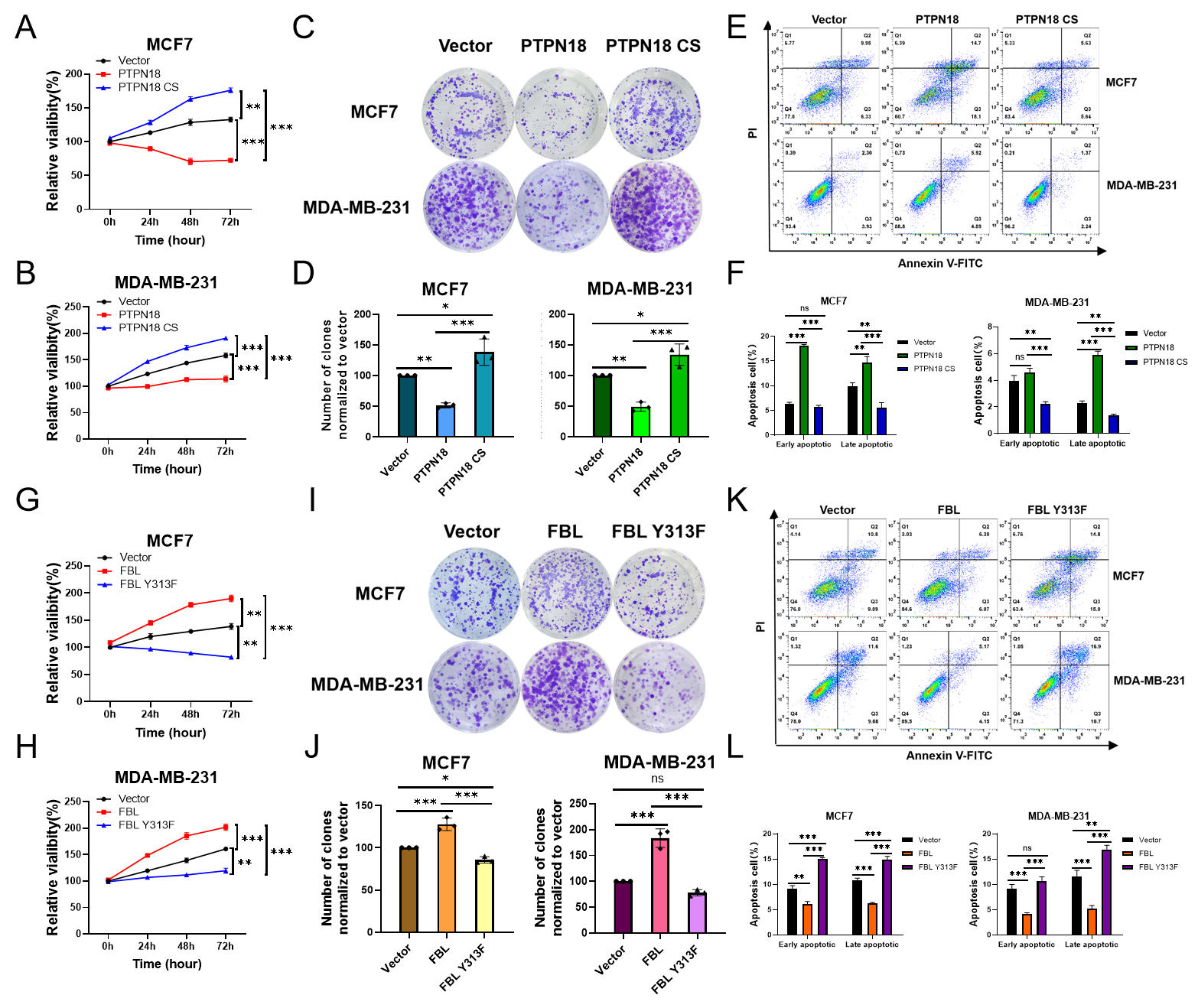

Supplement: Supplementary file 6 — Supplementary Figure 5 [file 41419_2025_8395_MOESM6_ESM.tif]

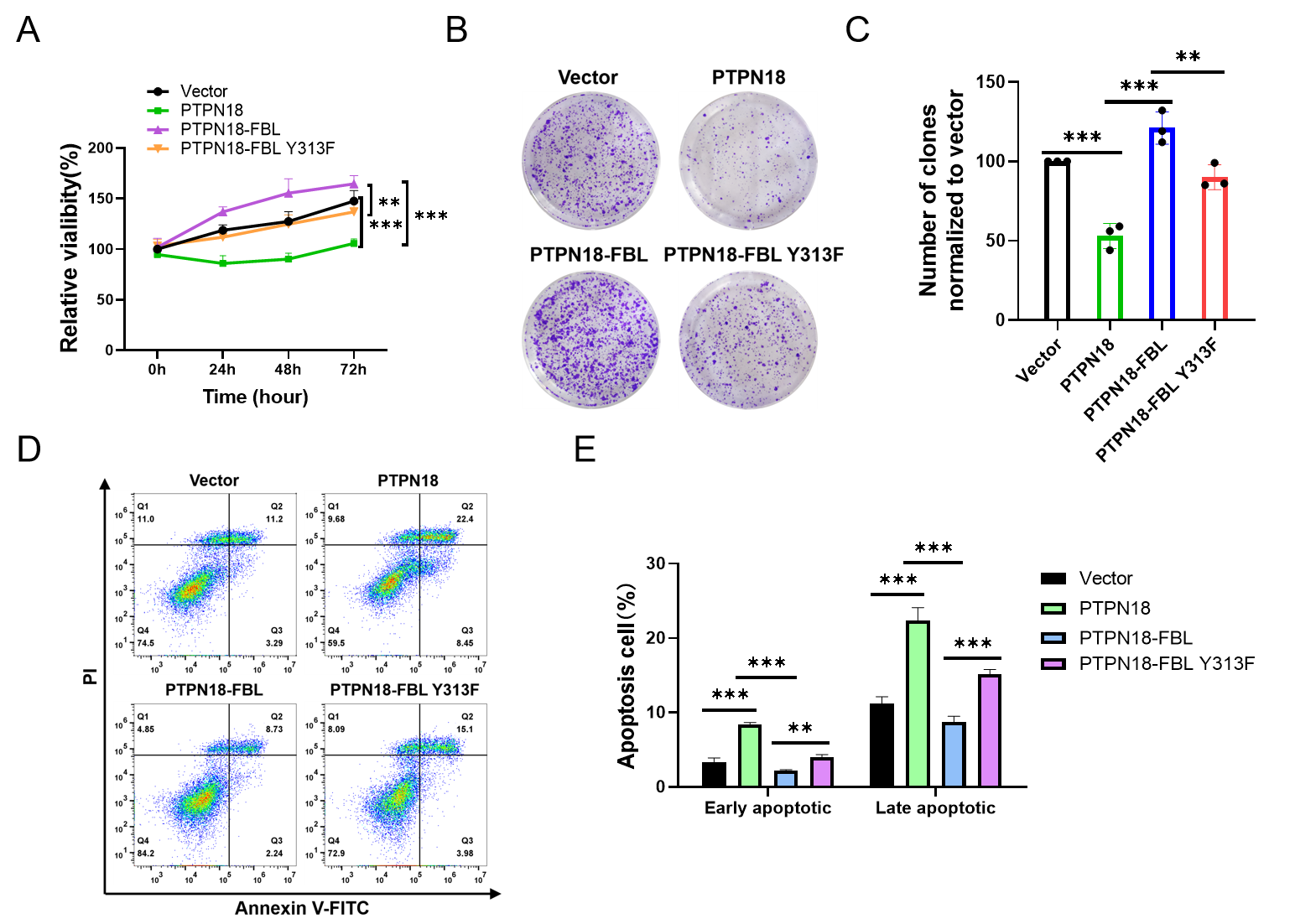

Supplement: Supplementary file 7 — Supplementary Figure 6 [file 41419_2025_8395_MOESM7_ESM.tif]

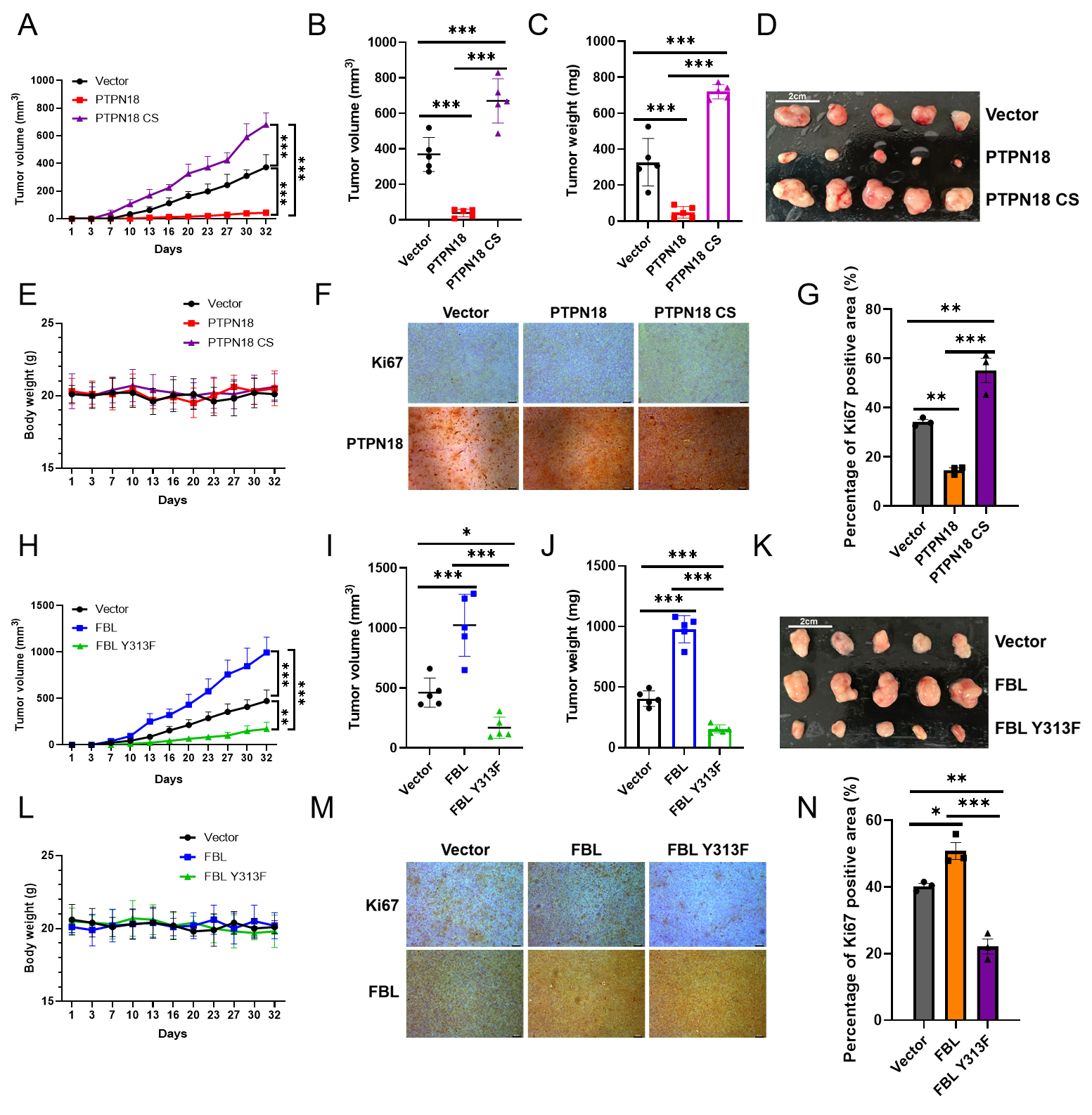

Supplement: Supplementary file 8 — Supplementary Figure 7 [file 41419_2025_8395_MOESM8_ESM.tif]

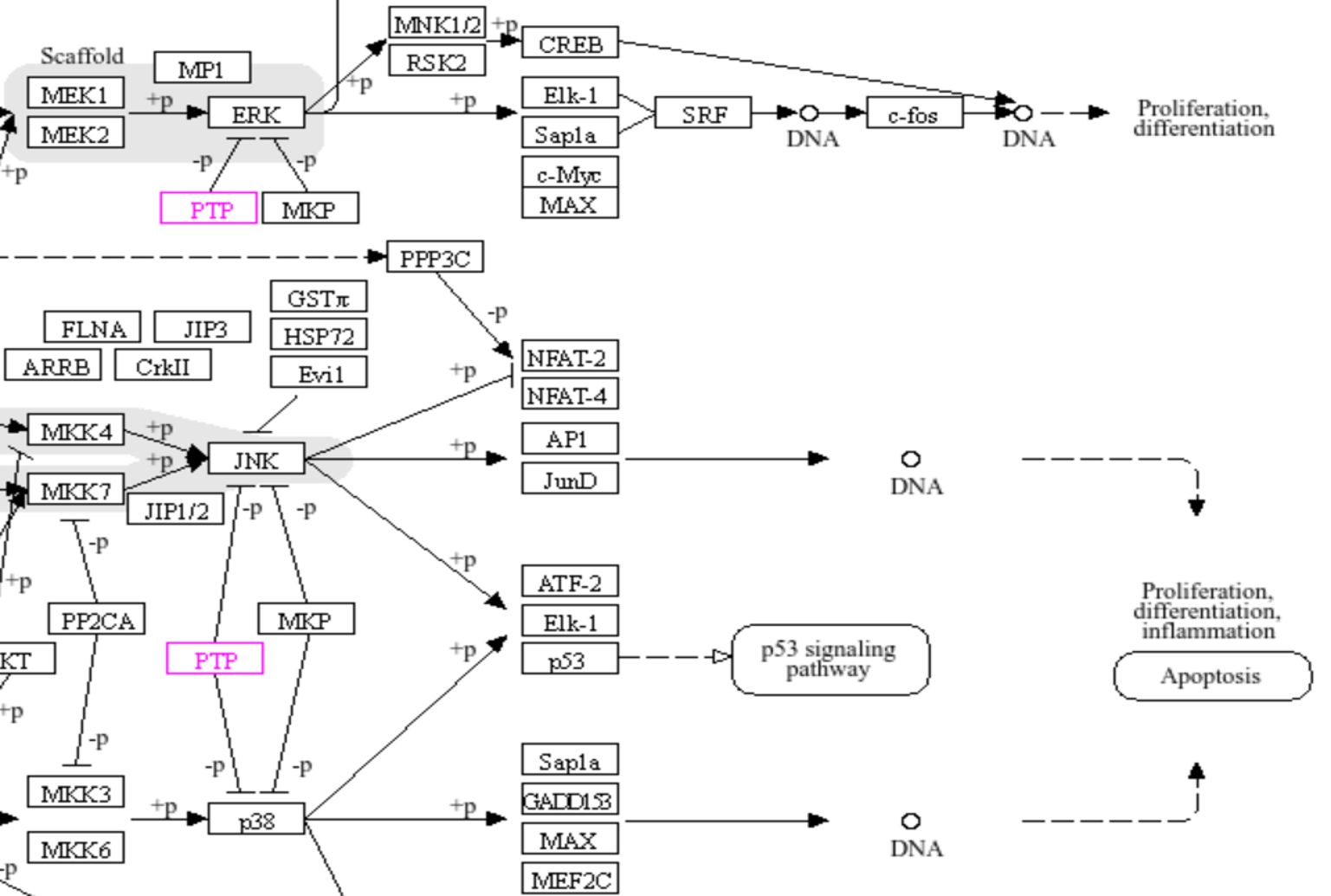

Supplement: Supplementary file 9 — Supplementary Figure 8 [file 41419_2025_8395_MOESM9_ESM.tif]
