# Supplementary material for: The R451 site is critical for PTPN18 to exert tumor suppressive effects in breast cancer through the negative regulatory interacting protein fibrillarin
Source: Cell Death Dis. 2026 Jan 20;17(1):168. doi: 10.1038/s41419-025-08395-1 (PMC12876892; doi:10.1038/s41419-025-08395-1)

Fig . 1C

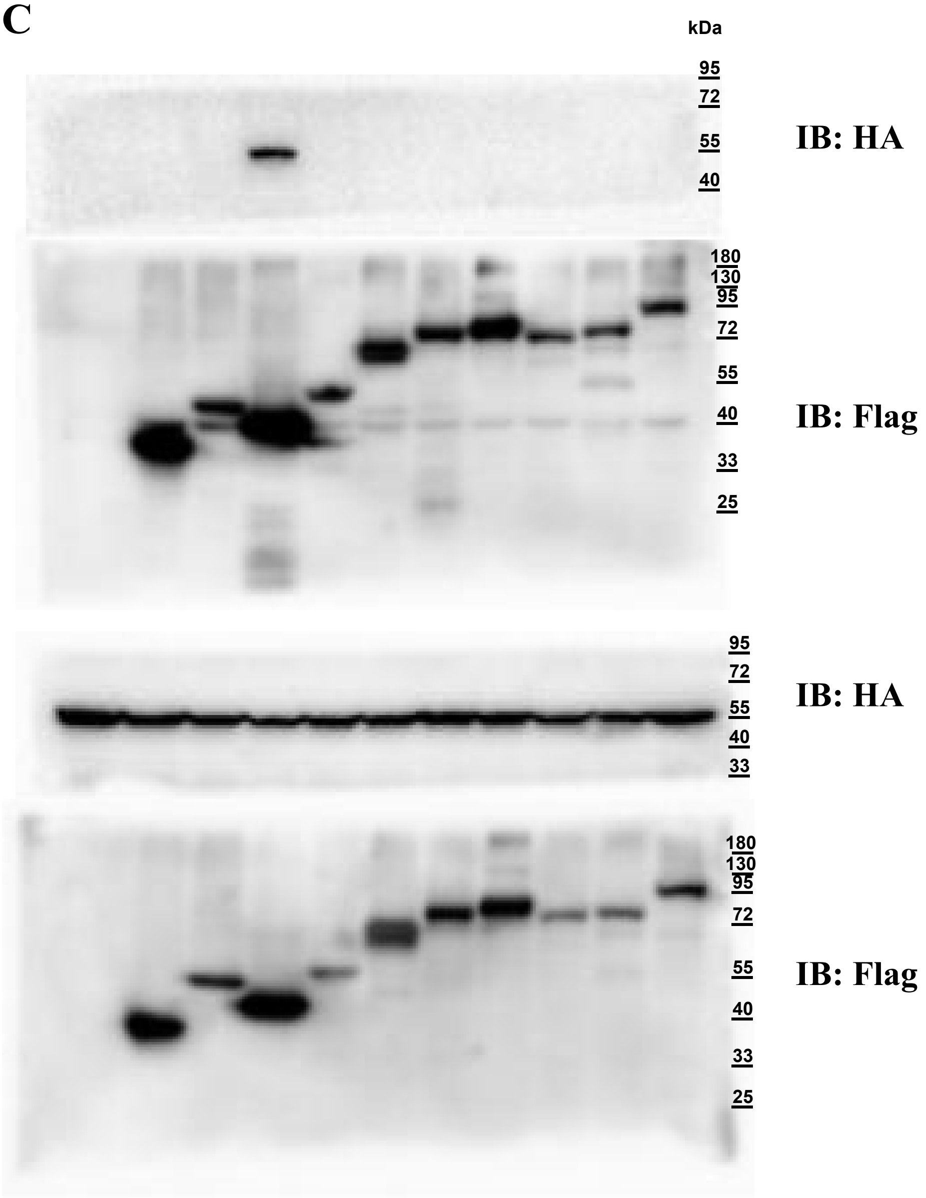

Fig . 1D

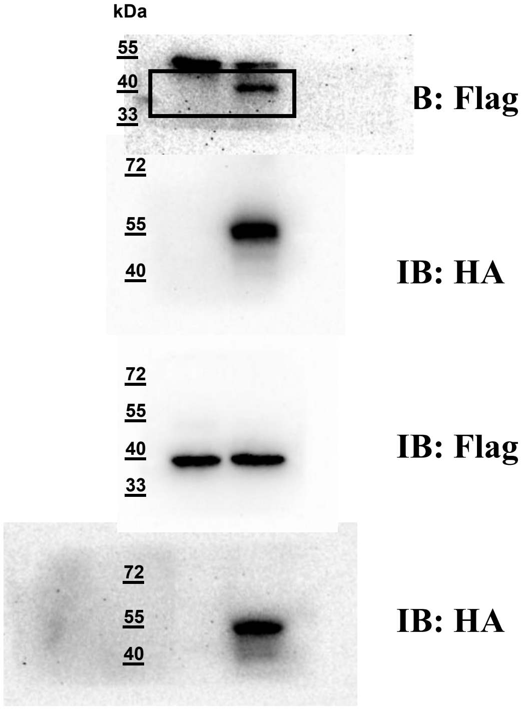

Fig . 1E

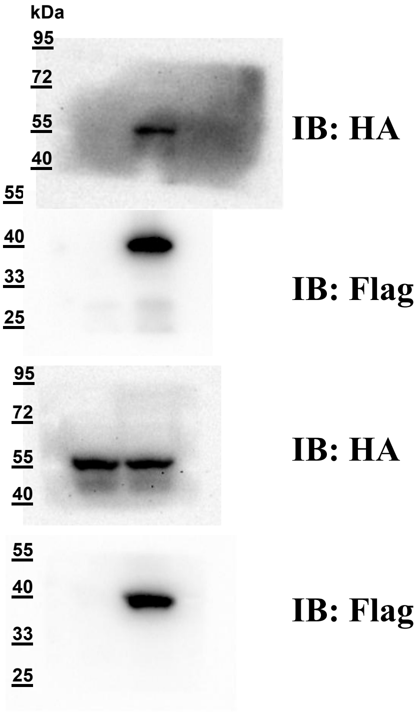

Fig . 1F

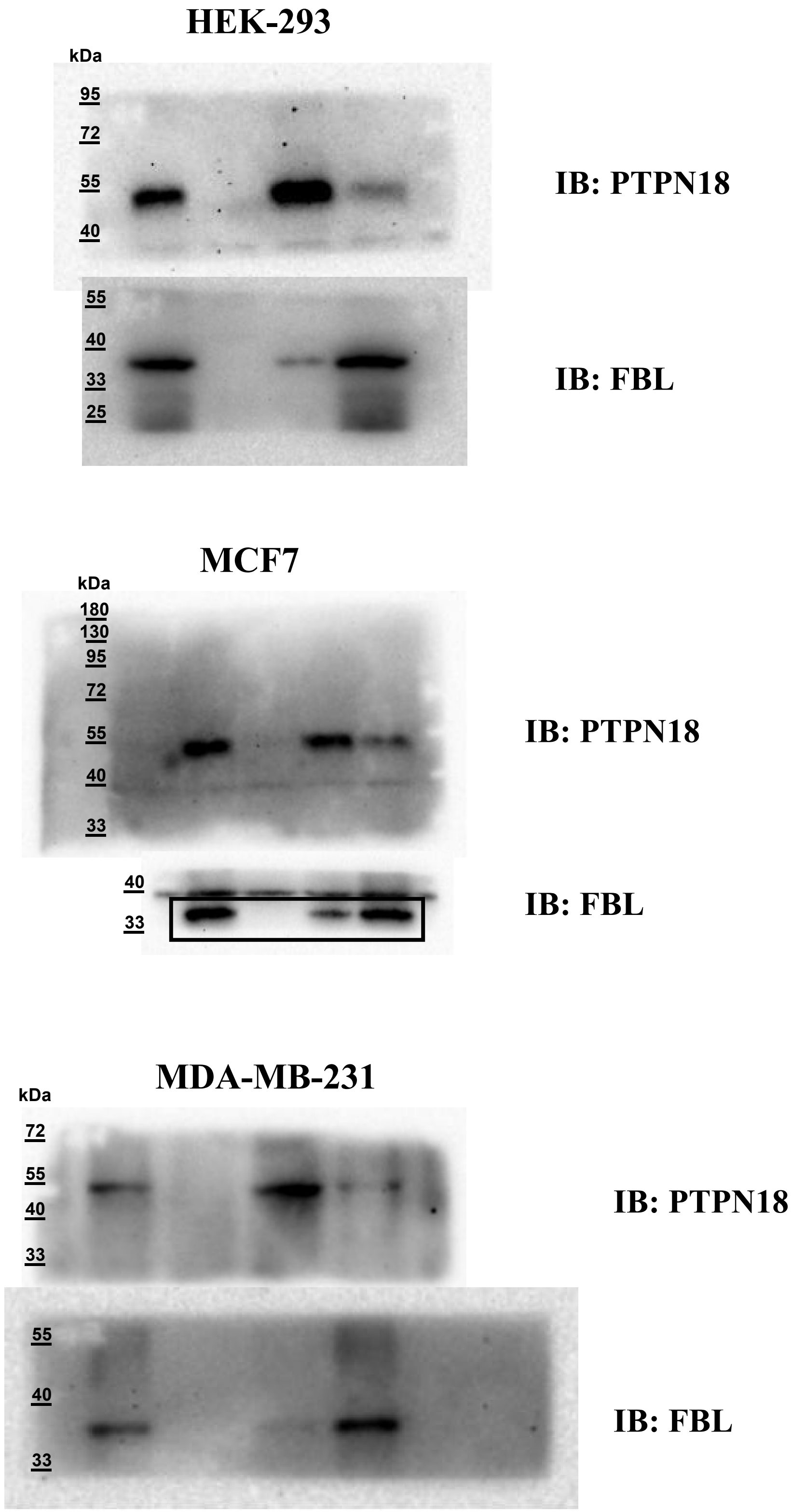

HEK-293
kDa
95
72
55
40
IB: PTPN18
55
40
33
25
IB: FBL
MCF7
kDa
180
130
95
72
55
40
33
IB: PTPN18
40
33
IB: FBL
MDA-MB-231
kDa
72
55
40
33
IB: PTPN18
55
40
33
IB: FBL

Fig . 2B

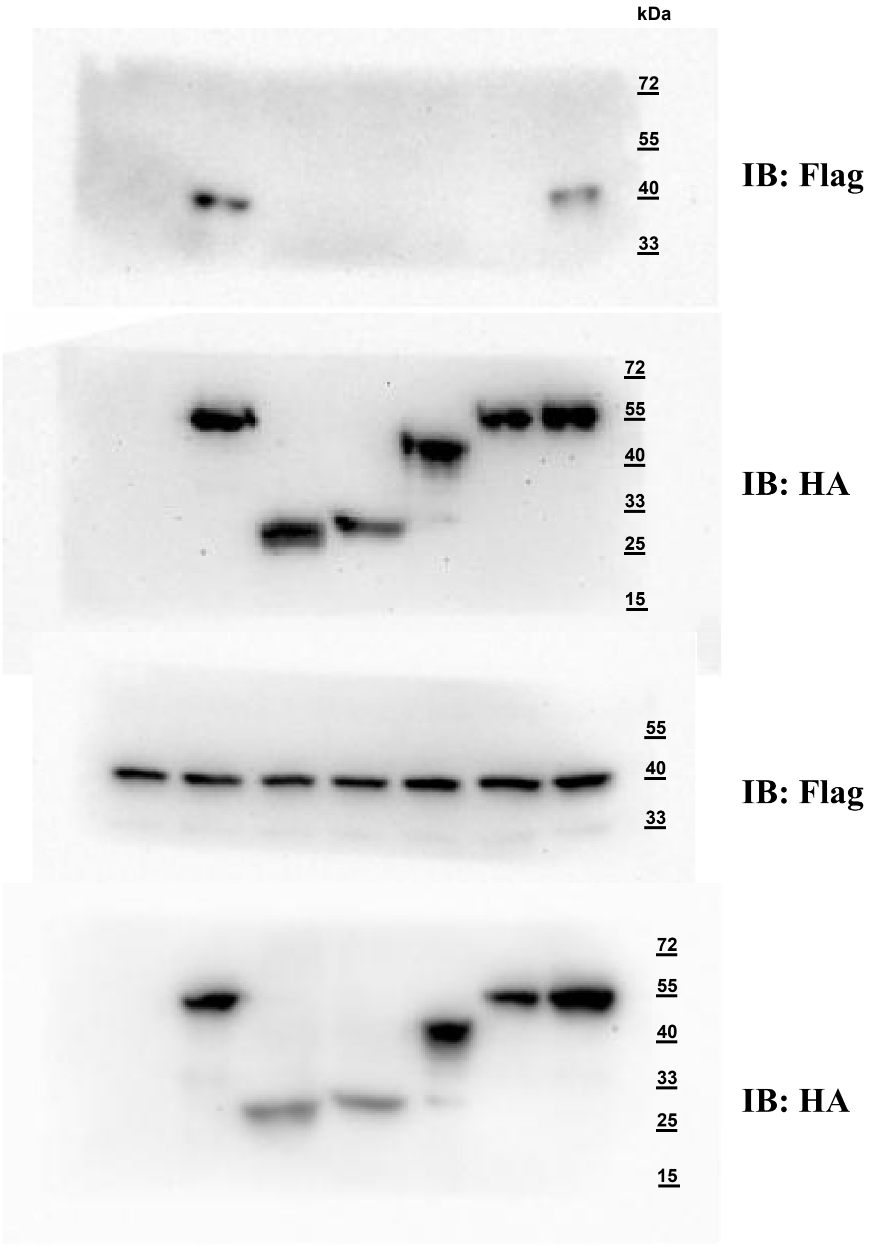
kDa
72
55
40
33
IB: Flag
72
55
40
33
25
15
IB: HA
55
40
33
IB: Flag
72
55
40
33
25
15
IB: HA

Fig . 2D

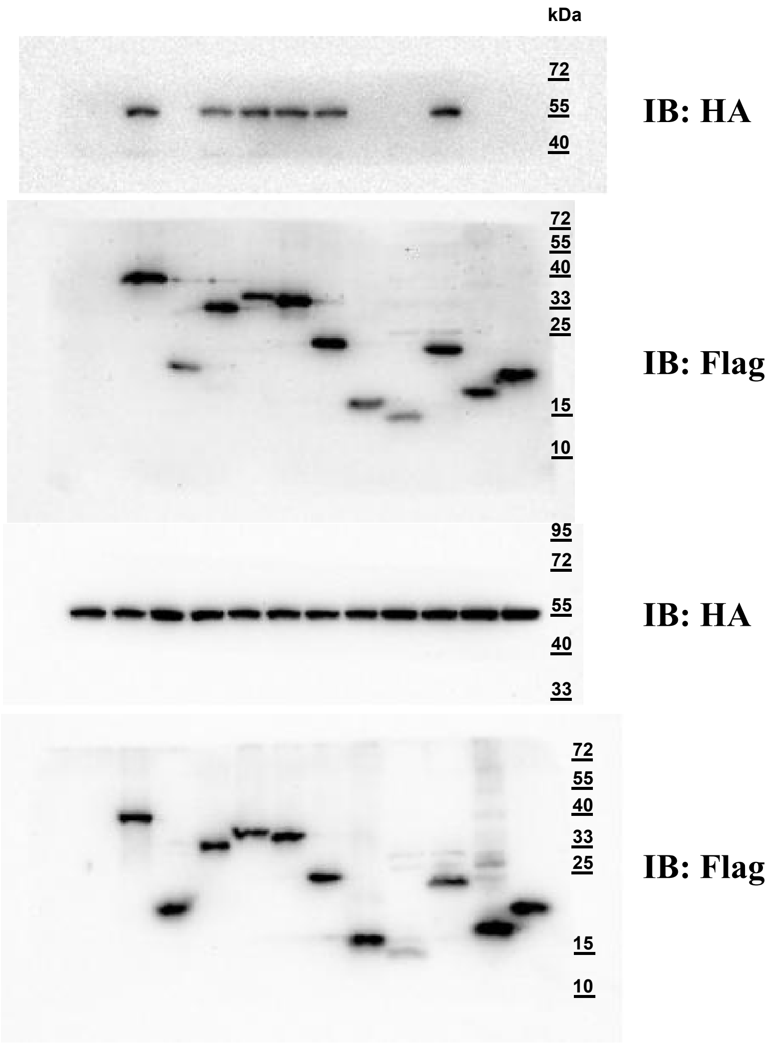
kDa
72
55
40
IB: HA
72
55
40
33
25
15
10
IB: Flag
95
72
55
40
33
IB: HA
72
55
40
33
25
15
10
IB: Flag

Fig . 2F

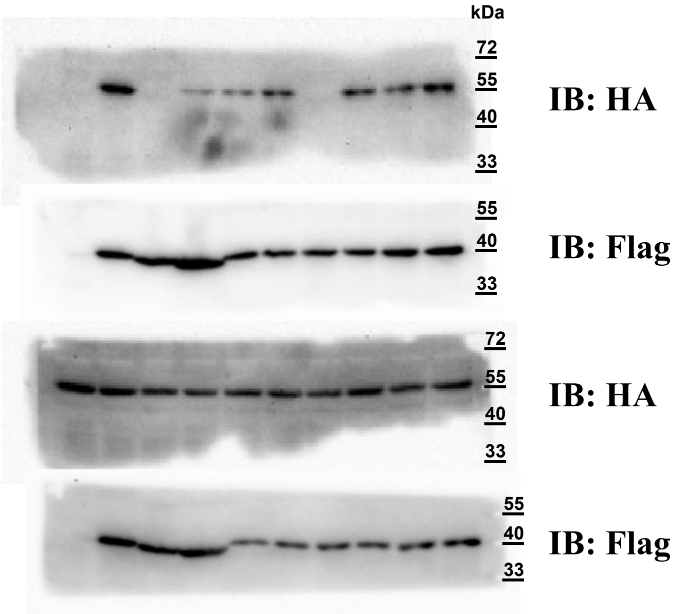
kDa
72
55
40
33
IB: HA
55
40
33
IB: Flag
72
55
40
33
IB: HA
55
40
33
IB: Flag

Fig . 3A

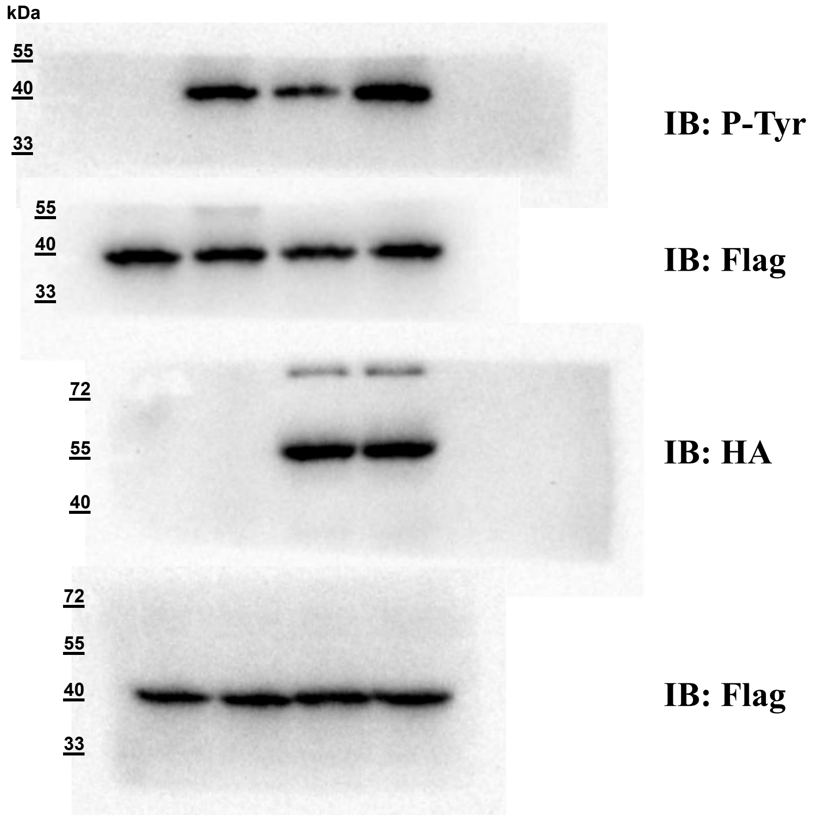
kDa
55
40
33
IB: P-Tyr
55
40
33
IB: Flag
72
55
40
IB: HA
72
55
40
33
IB: Flag

Fig . 3B

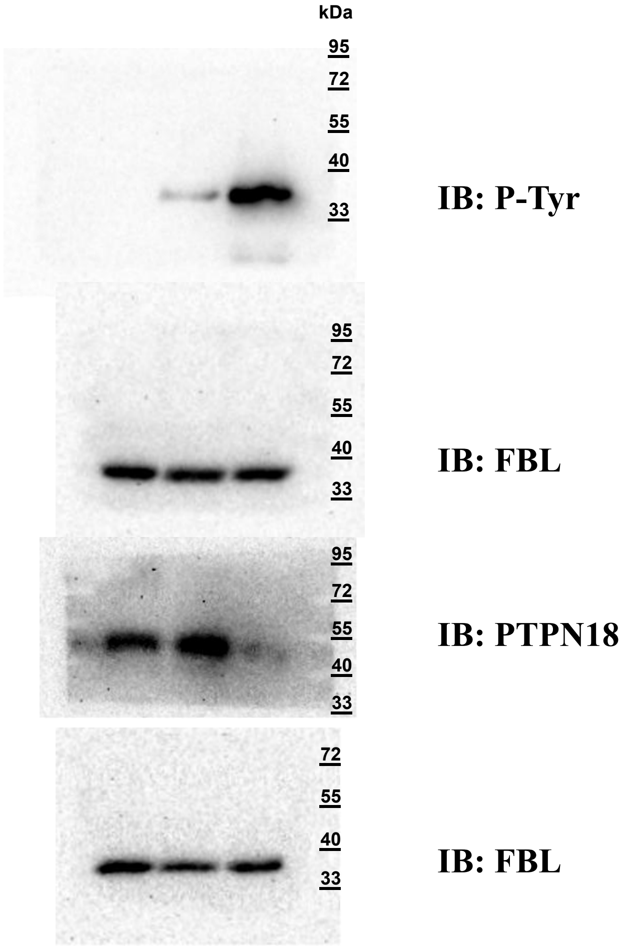
kDa
95
72
55
40
33
IB: P-Tyr
95
72
55
40
33
IB: FBL
95
72
55
40
33
IB: PTPN18
72
55
40
33
IB: FBL

Fig . 3C

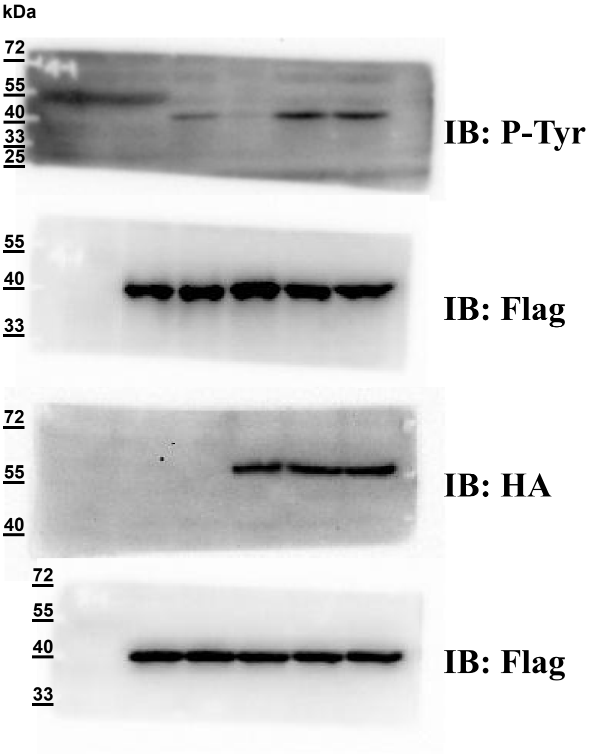

Fig . 3D

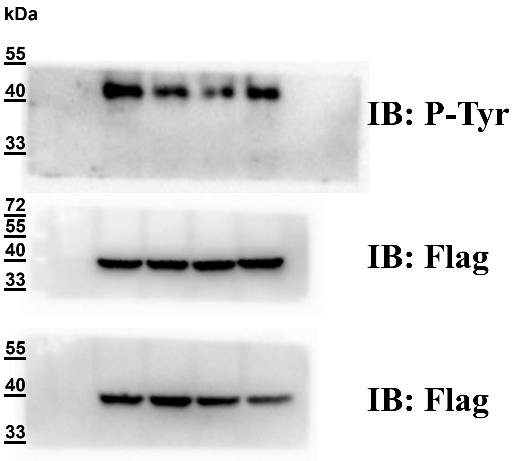

Fig . 3E

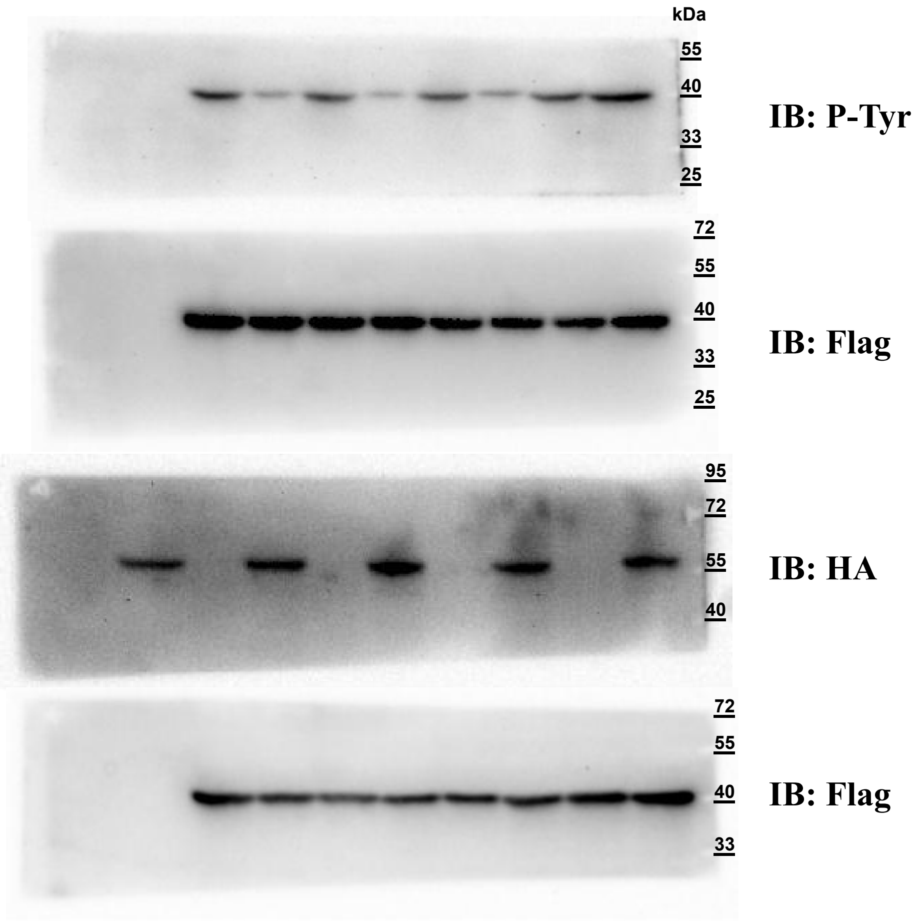

Fig . 4C

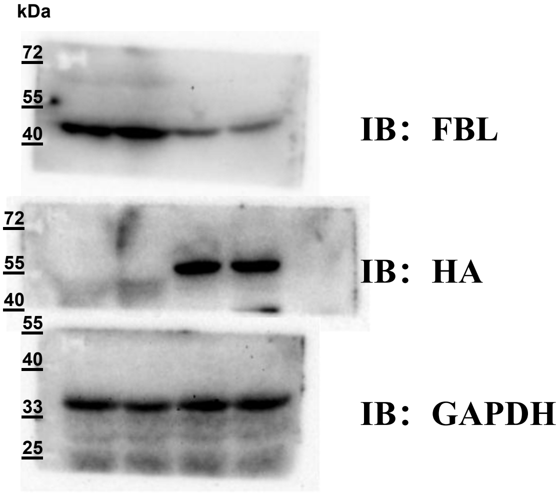

Fig . 4D

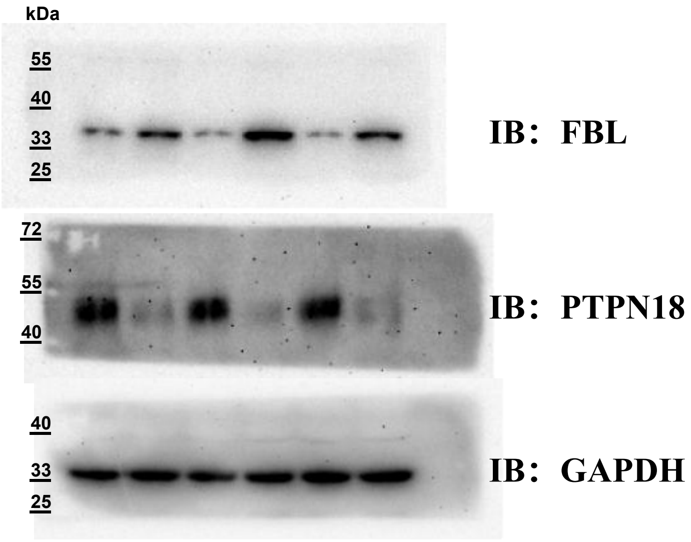

Fig . 4E

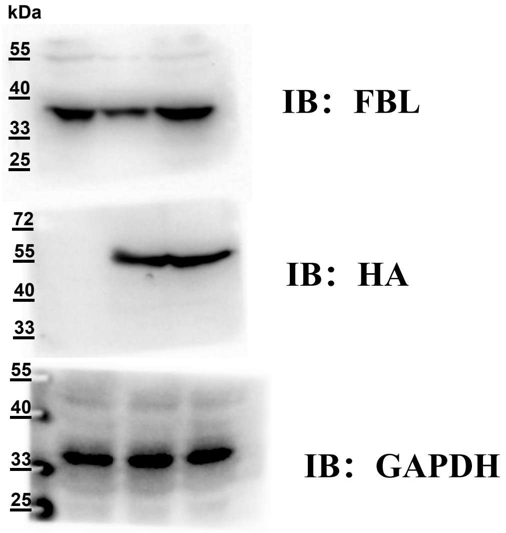

Fig . 4G

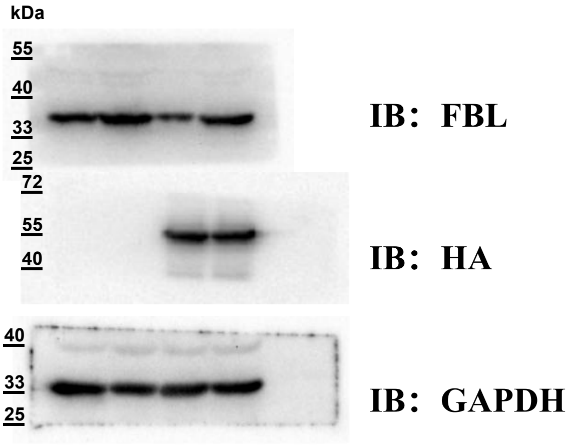

Fig . 4F

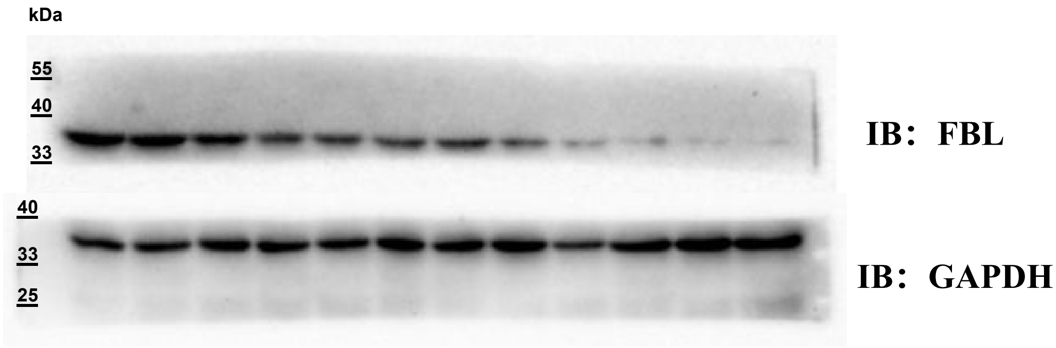

Fig . 4H

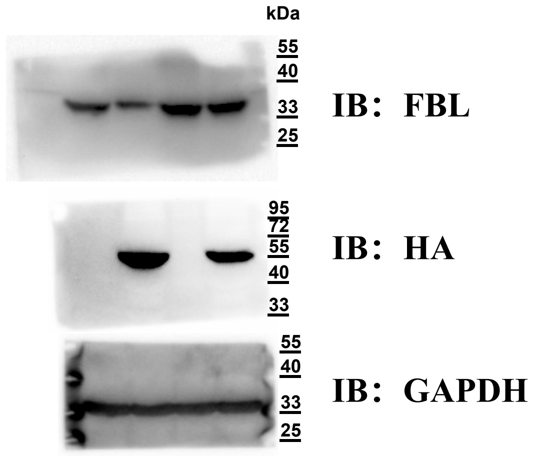

Fig . 4I

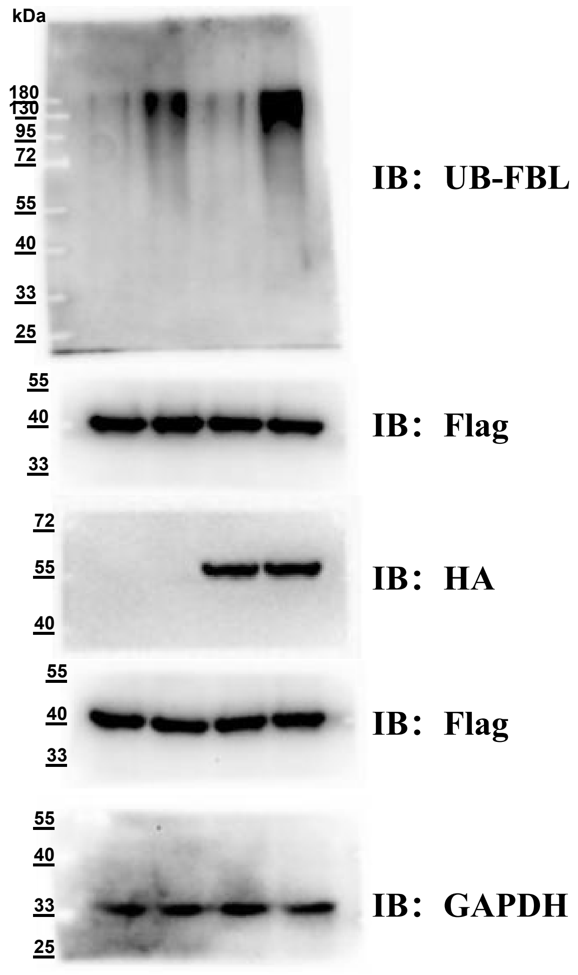

Fig . 4J

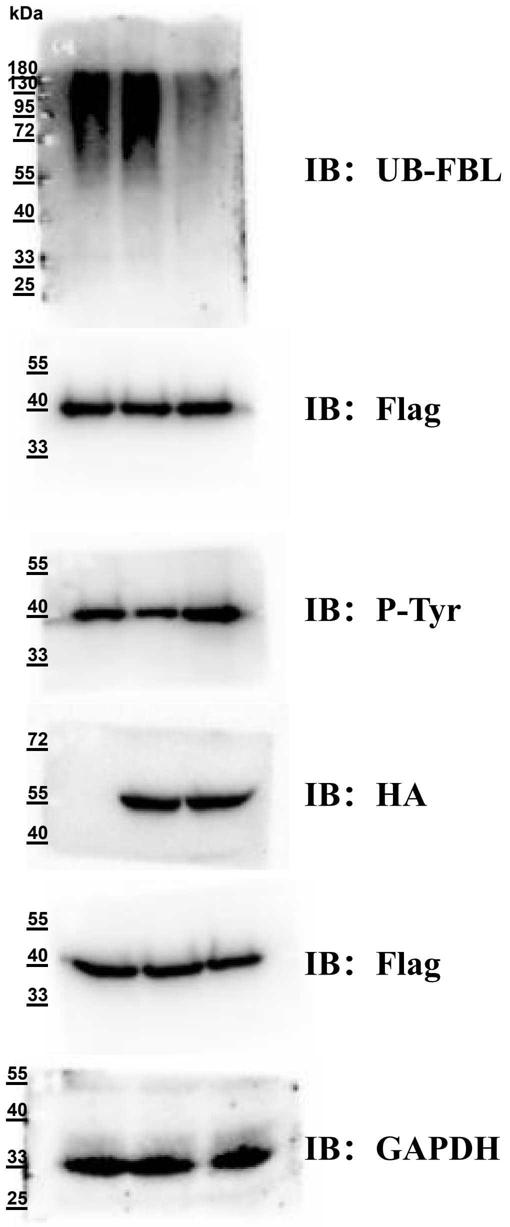

Fig . 4K

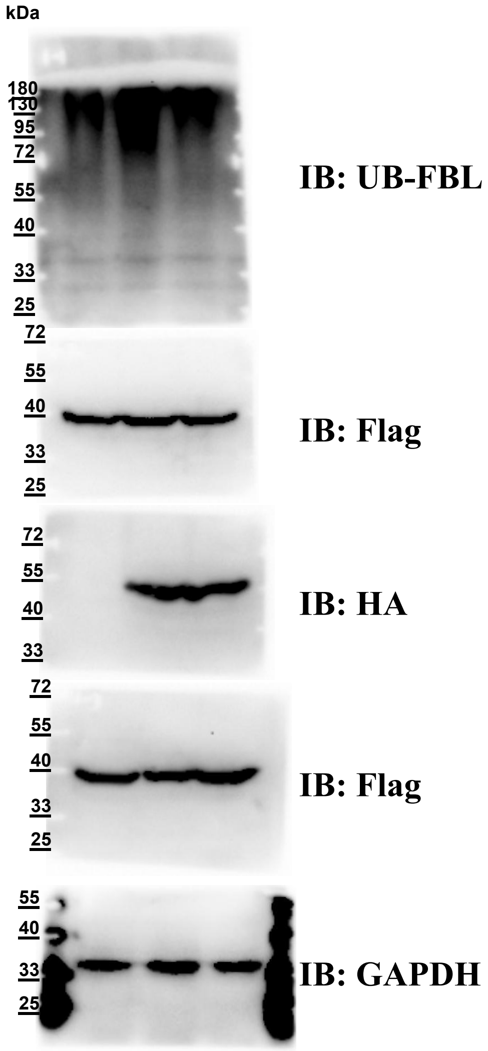
kDa
180
130
95
72
55
40
33
25
IB: UB-FBL
72
55
40
33
25
IB: Flag
72
55
40
33
IB: HA
72
55
40
33
25
IB: Flag
55
40
33
25
IB: GAPDH

Fig . 4L

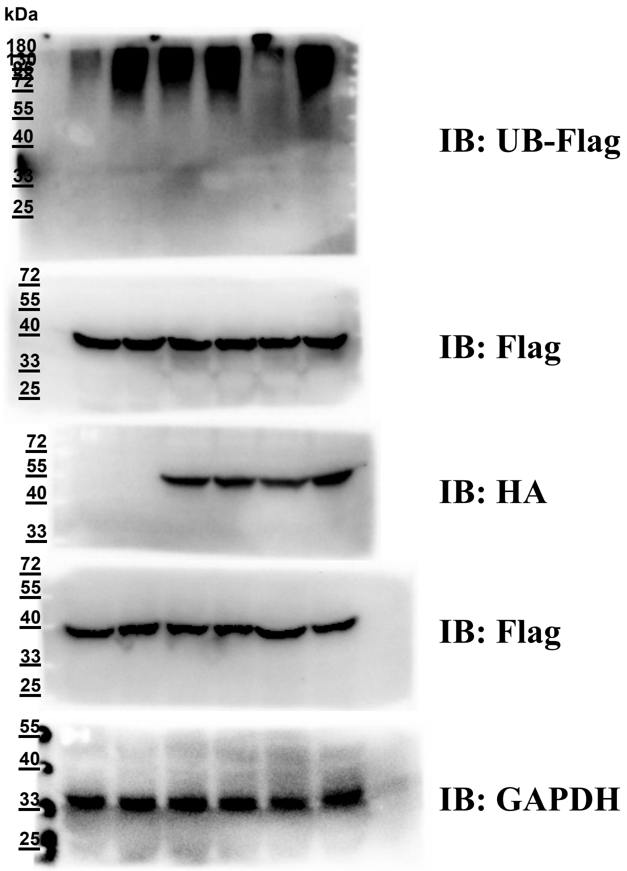
kDa
180
55
40
25
IB: UB-Flag
72
55
40
33
25
IB: Flag
72
55
40
33
IB: HA
72
55
40
33
25
IB: Flag
55
40
33
25
IB: GAPDH

Fig . 5C

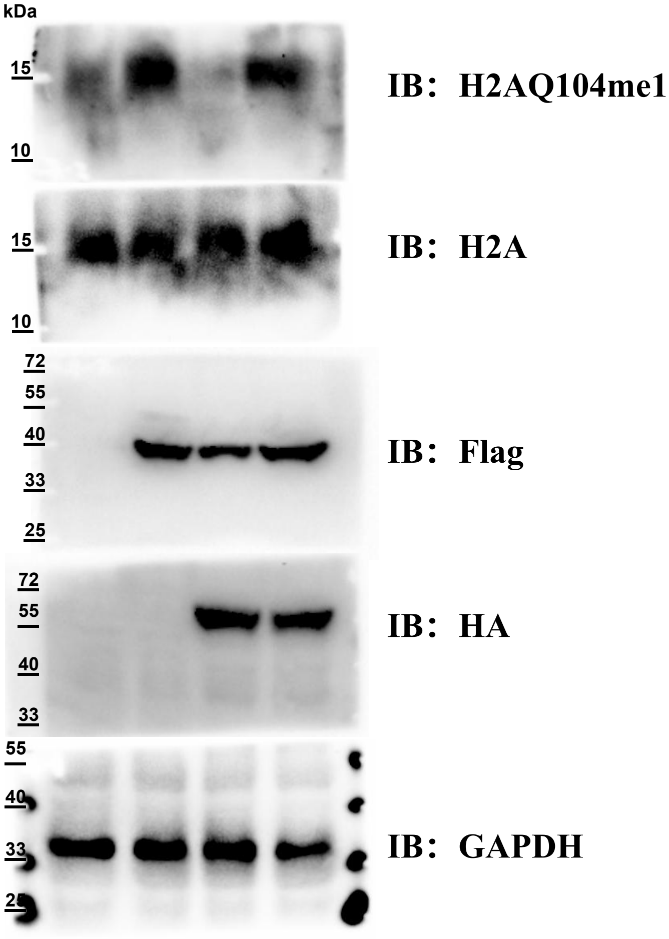
kDa
15
10
IB：H2AQ104me1
15
10
IB：H2A
72
55
40
33
25
IB：Flag
72
55
40
33
IB：HA
55
40
33
IB：GAPDH

Fig . 5E

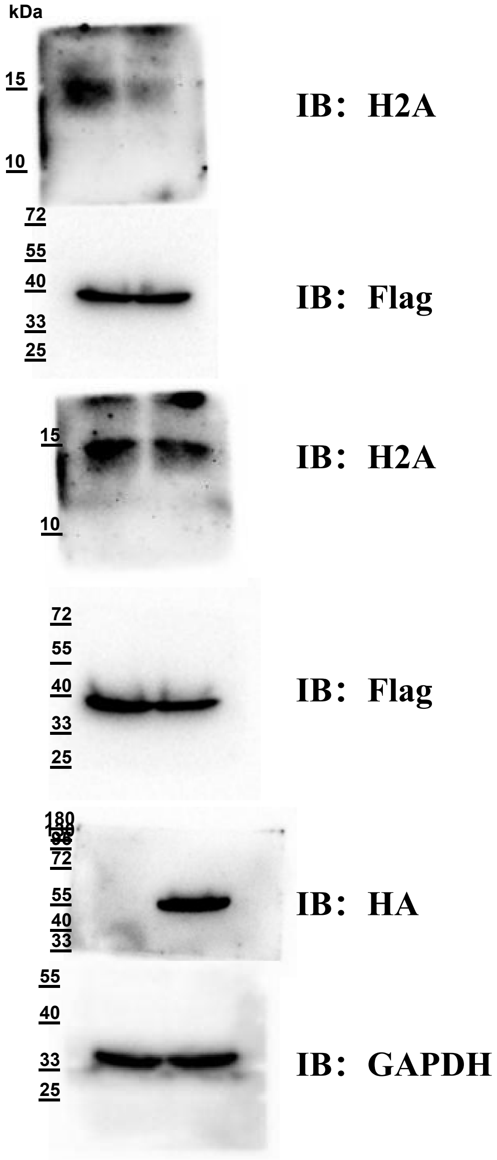
kDa
15
10
IB：H2A
72
55
40
33
25
IB：Flag
15
10
IB：H2A
72
55
40
33
25
IB：Flag
180
72
55
40
33
IB：HA
55
40
33
25
IB：GAPDH

Fig . 8A

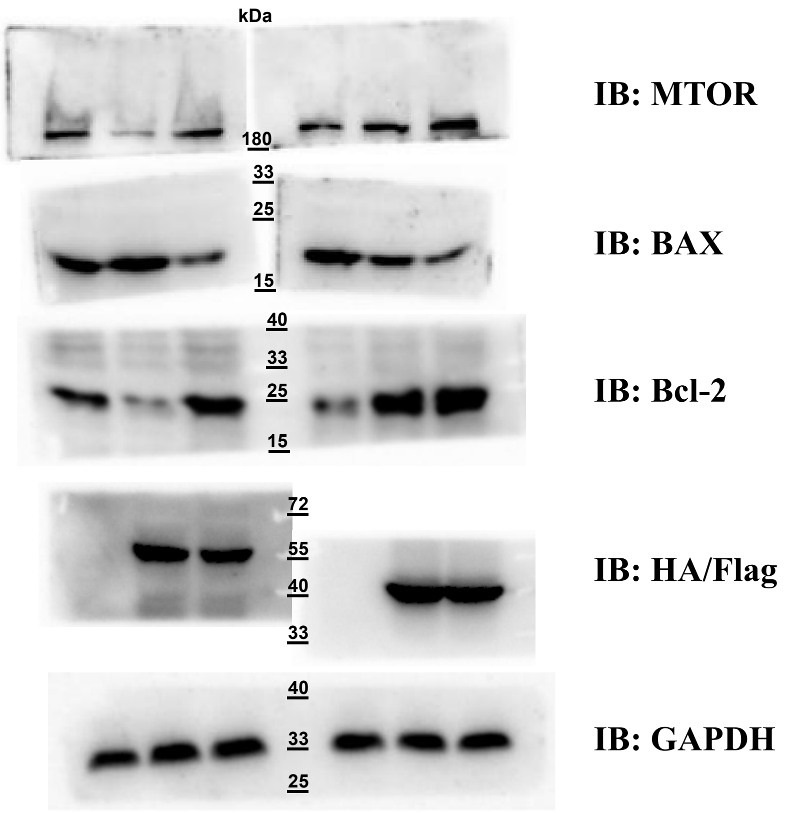

kDa
IB: MTOR
180
33
25
IB: BAX
15
40
33
25
IB: Bcl-2
15
72
55
IB: HA/Flag
40
33
40
IB: GAPDH
33
25

Fig . 8C

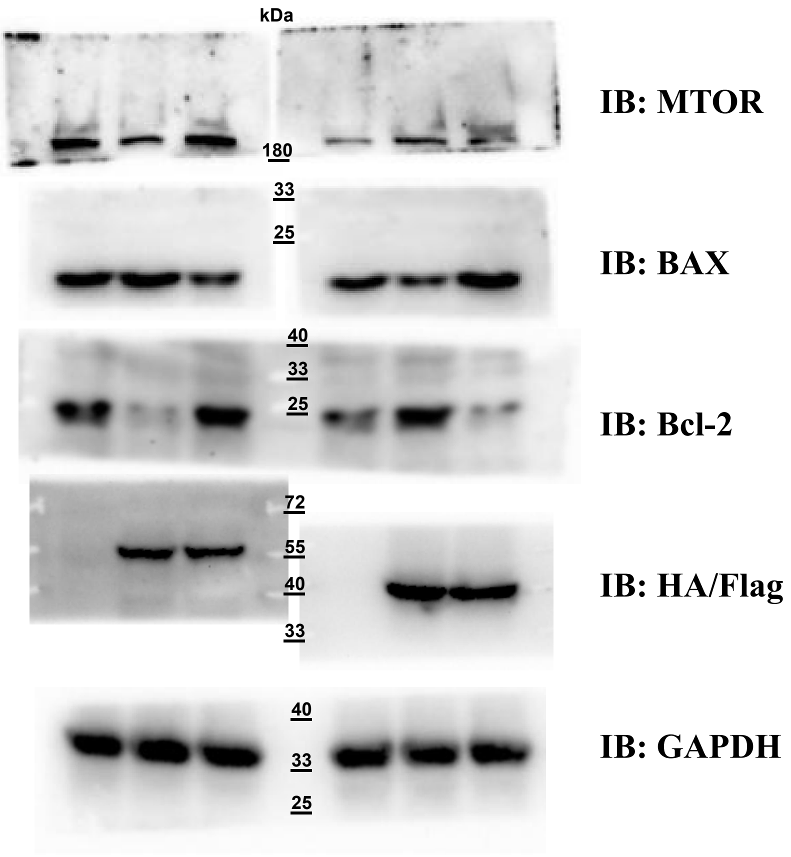

kDa
IB: MTOR
180
33
25
IB: BAX
40
33
25
IB: Bcl-2
72
55
IB: HA/Flag
40
33
40
IB: GAPDH
33
25

Fig . 8E

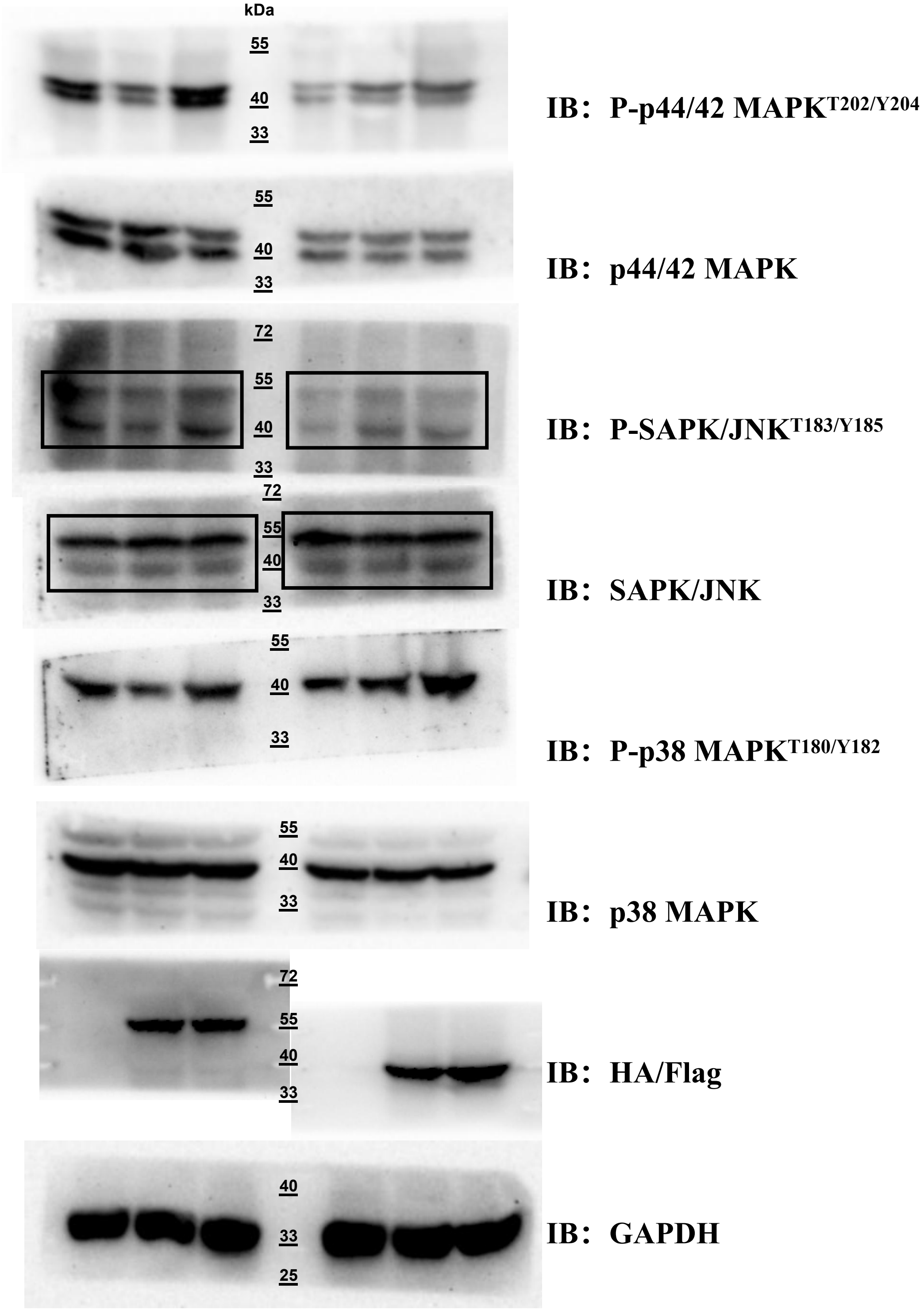
kDa
55
40
33
IB: P-p44/42 MAPK$^{T202/Y204}$
55
40
33
IB: p44/42 MAPK
72
55
40
33
IB: P-SAPK/JNK$^{T183/Y185}$
72
55
40
33
IB: SAPK/JNK
55
40
33
IB: P-p38 MAPK$^{T180/Y182}$
55
40
33
IB: p38 MAPK
72
55
40
33
IB: HA/Flag
40
33
25
IB: GAPDH

Fig . 8G

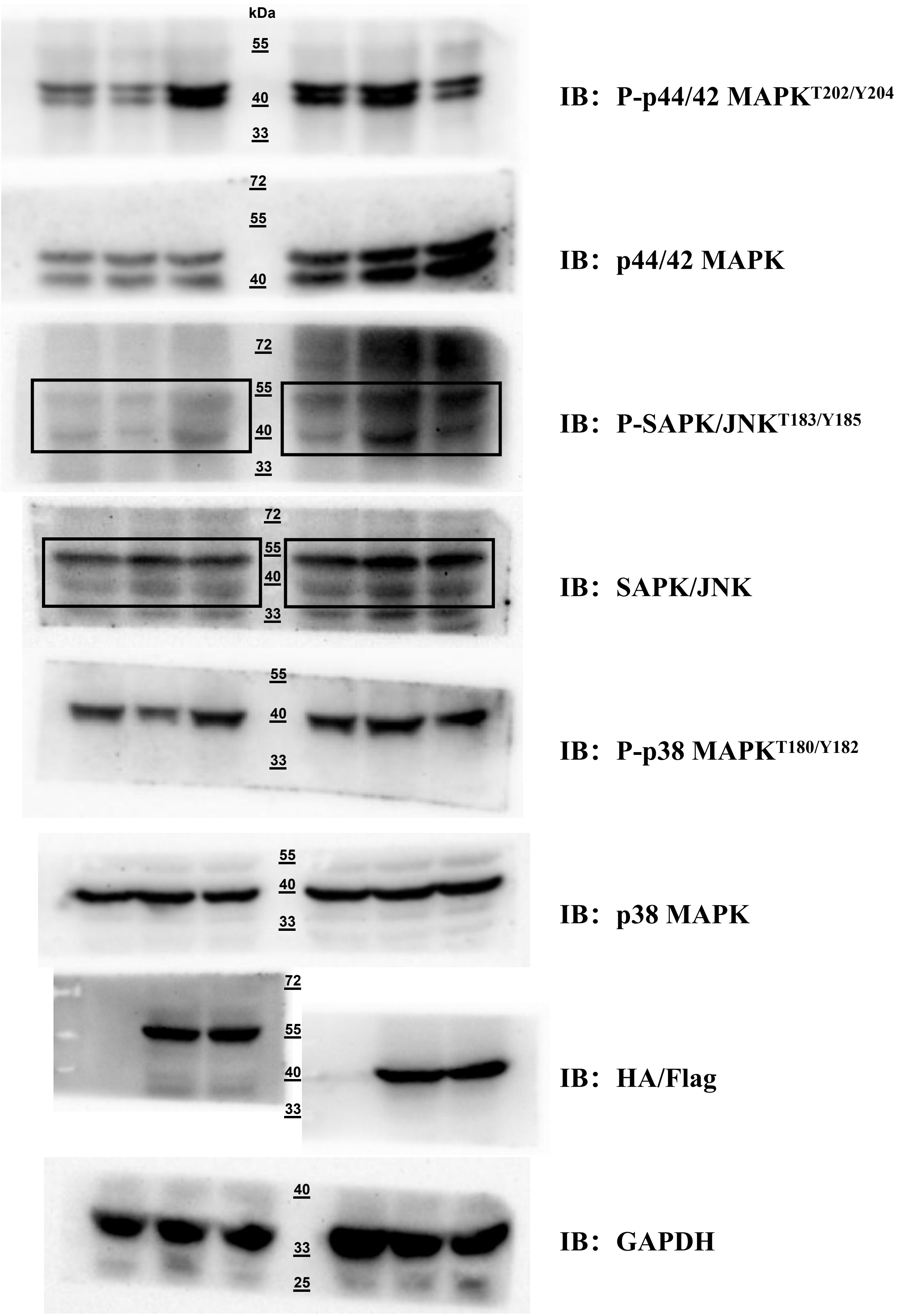
kDa
55
40
33
IB: P-p44/42 MAPK$^{T202/Y204}$
72
55
40
IB: p44/42 MAPK
72
55
40
33
IB: P-SAPK/JNK$^{T183/Y185}$
72
55
40
33
IB: SAPK/JNK
55
40
33
IB: P-p38 MAPK$^{T180/Y182}$
55
40
33
IB: p38 MAPK
72
55
40
33
IB: HA/Flag
40
33
25
IB: GAPDH

Fig . S2A

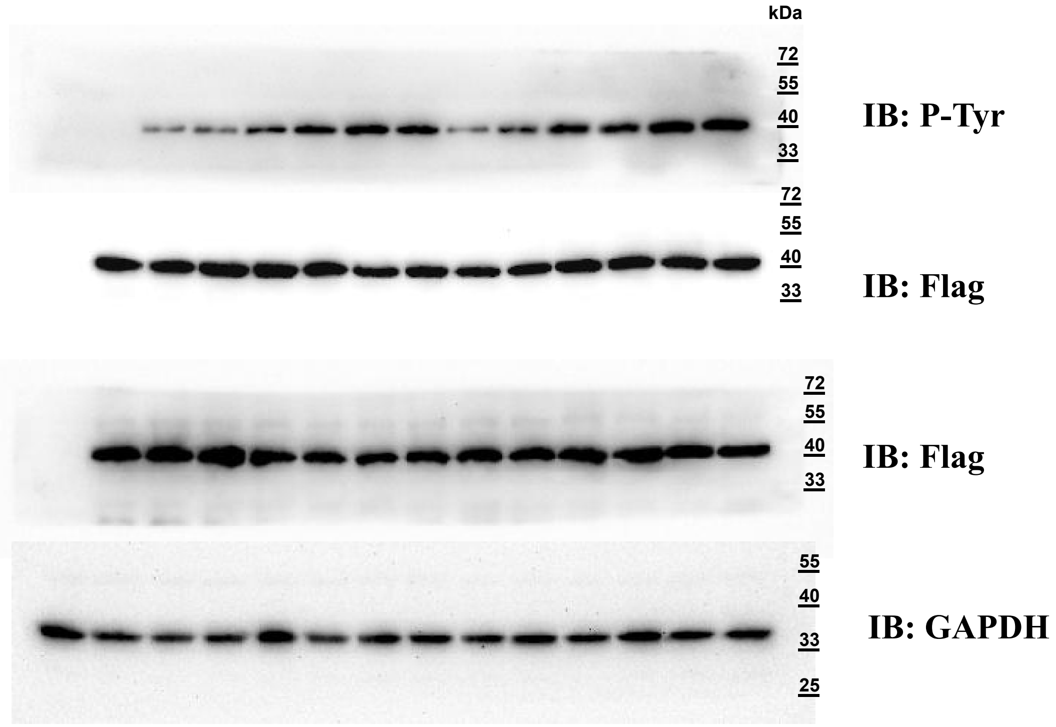

Fig . S2B

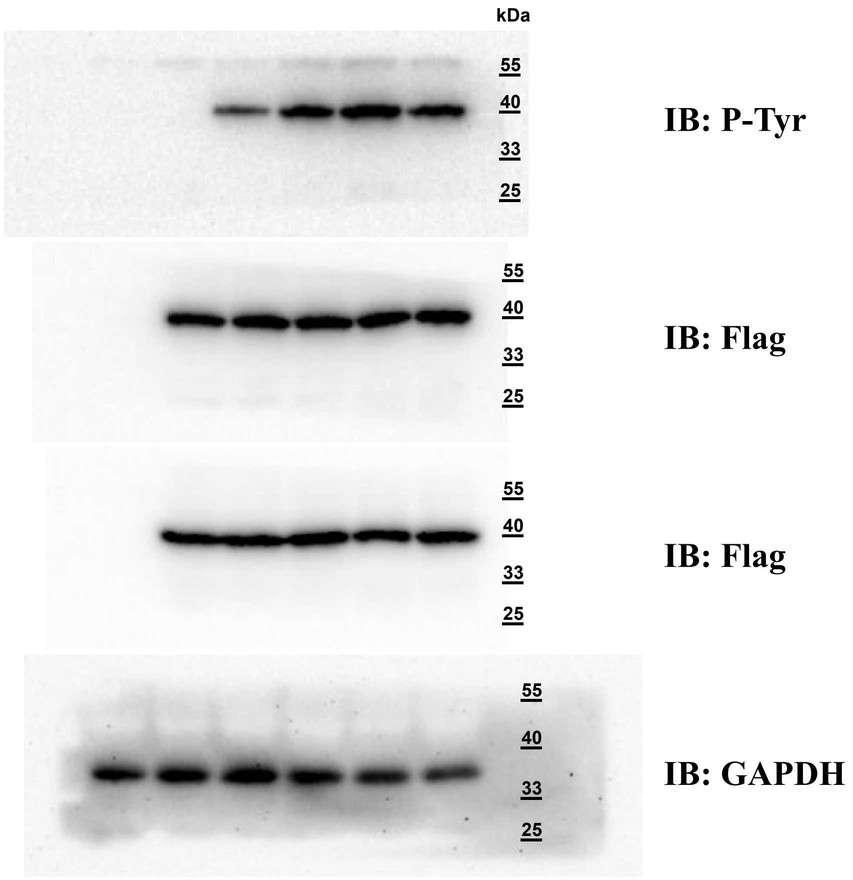

Fig . S3A

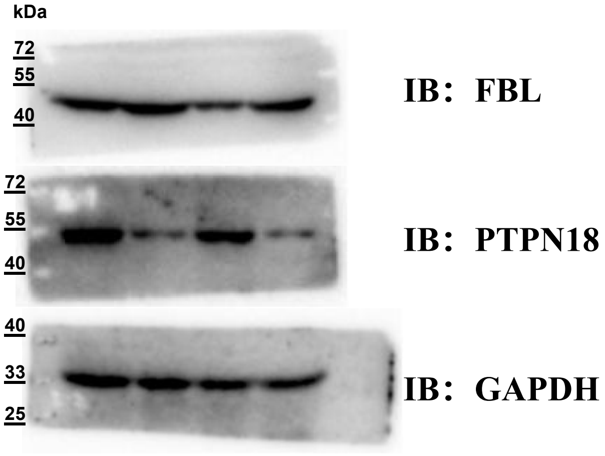

Fig . S3B

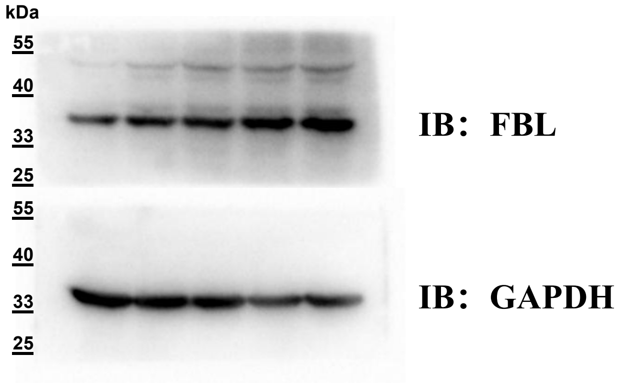

Fig . S3C

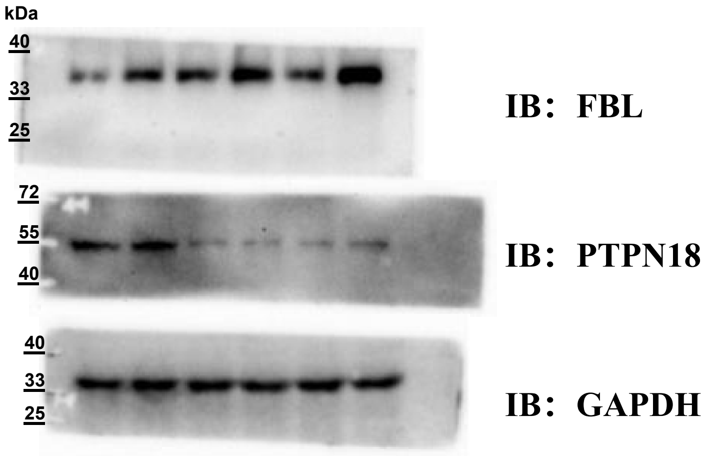

Fig . S3D

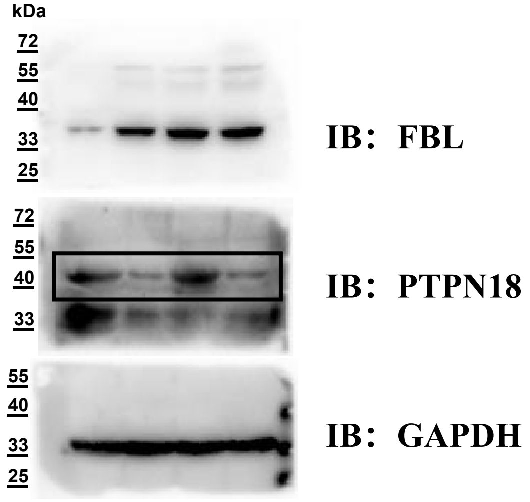

Fig . S3E

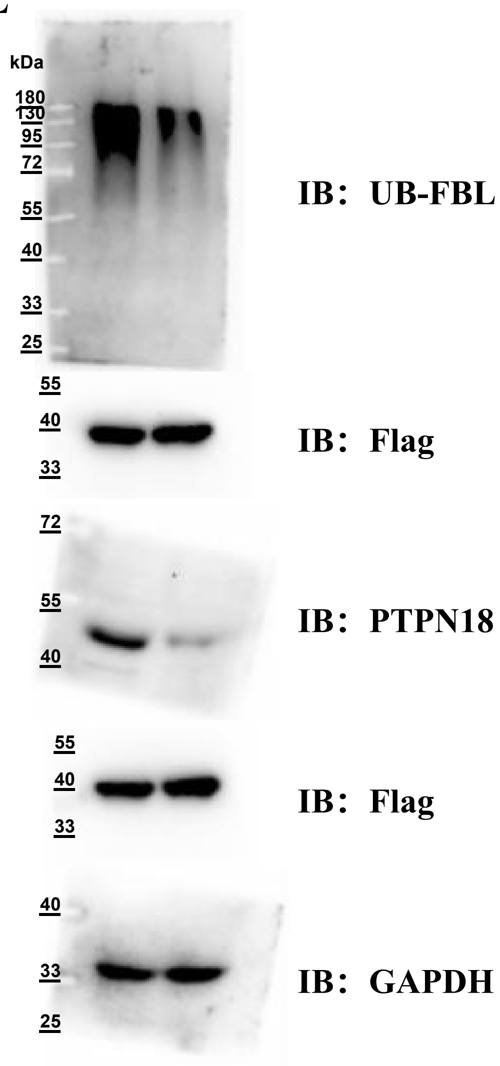

Fig . S4C

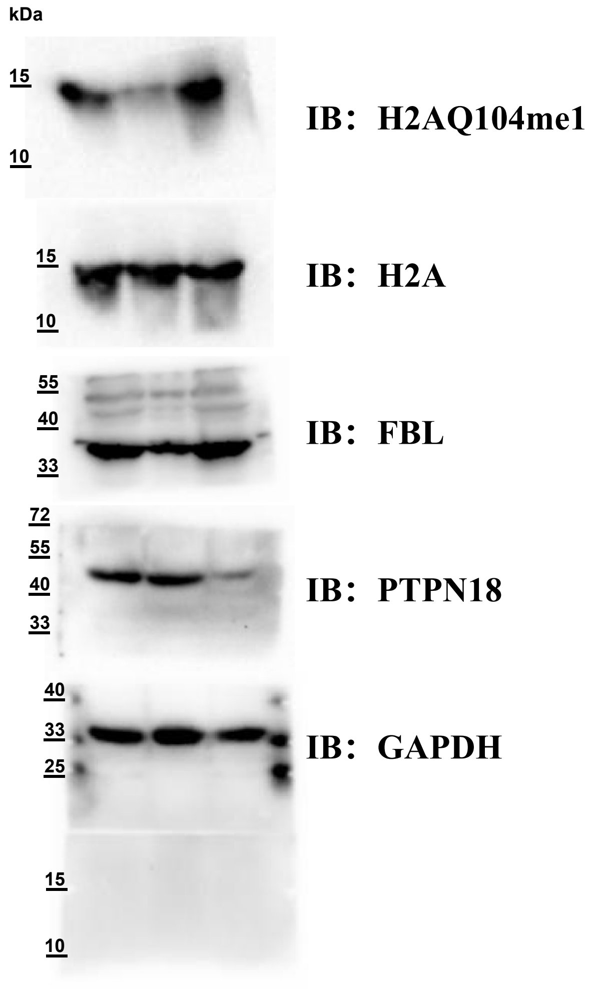

Fig . S4E

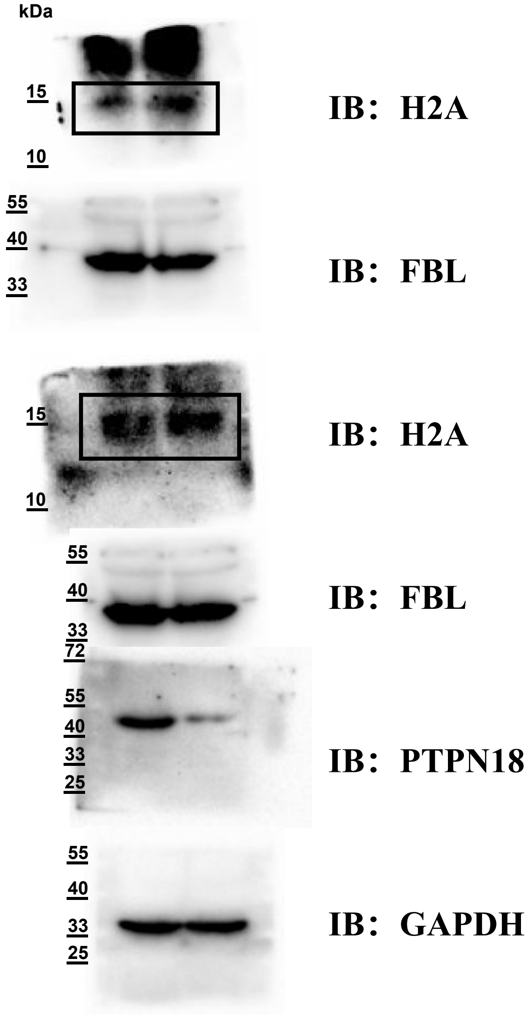

Supplement: Supplementary file 11 — Original Western Data [file 41419_2025_8395_MOESM11_ESM.pdf]
